# Coenzyme A Restriction as a Factor Underlying Pre-Eclampsia with Polycystic Ovary Syndrome as a Risk Factor

**DOI:** 10.3390/ijms23052785

**Published:** 2022-03-03

**Authors:** Charlie Hodgman, Gulafshana Hafeez Khan, William Atiomo

**Affiliations:** 1 School of Biosciences, University of Nottingham, Loughborough LE12 5RD, UK; 2 Department of Gynaecology and Obstetrics, Maroof International Hospital, Islamabad 04412, Pakistan; gulafshanahafeez@hotmail.com; 3 Lifespan and Population Health, School of Medicine, University of Nottingham, Nottingham NG7 2UH, UK; 4 College of Medicine, Mohammed Bin Rashid University of Medical and Health Sciences, Dubai Healthcare City, Dubai 505055, United Arab Emirates

**Keywords:** pre-eclampsia, polycystic ovary syndrome, adverse antenatal conditions, Coenzyme A, metabolomics, transcriptomics, genome-wide association study, placenta, physiological dysregulation, systems pathology

## Abstract

Pre-eclampsia is the most common pregnancy complication affecting 1 in 20 pregnancies, characterized by high blood pressure and signs of organ damage, most often to the liver and kidneys. Metabolic network analysis of published lipidomic data points to a shortage of Coenzyme A (CoA). Gene expression profile data reveal alterations to many areas of metabolism and, crucially, to conflicting cellular regulatory mechanisms arising from the overproduction of signalling lipids driven by CoA limitation. Adverse feedback loops appear, forming sphingosine-1-phosphate (a cause of hypertension, hypoxia and inflammation), cytotoxic isoketovaleric acid (inducing acidosis and organ damage) and a thrombogenic lysophosphatidyl serine. These also induce mitochondrial and oxidative stress, leading to untimely apoptosis, which is possibly the cause of CoA restriction. This work provides a molecular basis for the signs of pre-eclampsia, why polycystic ovary syndrome is a risk factor and what might be done to treat and reduce the risk of disease.

## 1. Introduction

Pre-eclampsia is a significant cause of maternal and foetal morbidity and mortality, accounting for at least 63,000 maternal deaths per annum [1]. Affected women usually present with new-onset hypertension and proteinuria developing during the second half of pregnancy, though presentation can vary. Systemic inflammation, thrombogenesis and acidosis may also occur. In severe cases, organ damage to the kidney and liver occurs, with haematological involvement and, if left to progress, women go on to experience neurological complications including seizures or eclamptic fits. The babies of women with pre-eclampsia are at increased risk of intra-uterine growth retardation, placental abruption, pre-term rupture of membranes, premature delivery and death.

Although the exact cause of pre-eclampsia (PE) is unknown, disordered placentation during early trophoblastic invasion of the maternal placental bed is a key feature [2]. It is thought to lead to vasoconstriction, poor blood supply to the growing foetus and release of various systemic factors into maternal circulation in response to hypoxic and oxidative stress of the syncytiotrophoblast [3]. However, there remain gaps in our understanding of the pathophysiology of the disease. For example, it has been suggested that there are two subtypes: early onset arising from disordered placentation and late onset (with a watershed of 34 weeks’ gestation) thought to arise from a maternal genetic predisposition to cardiovascular and metabolic disease [4]. 

Omics’ techniques are increasingly being used to identify potential biomarkers and molecular features of various disease states. A systematic review with meta-analysis of normal and pre-eclamptic placental gene expression has identified changes in numerous regulatory pathways [5]. Metabolomics aims to identify the entire spectrum of metabolites in a sample but requires multiple laboratory protocols and spectroscopic techniques to approach a complete profile. Proteomics data are less useful because disruption of the syncytiotrophoblast results in intracellular proteins masking any potential signalling molecules [3]. This also makes interpretation of biomarkers in common between PCOS and PE difficult to interpret [6]. An integrative approach is therefore needed in order to understand the principles underlying the metabolic regulation of a system and how their combined interactions are associated with variation in clinical phenotypes and resulting pathophysiology [7]. 

Polycystic ovarian syndrome (PCOS) is the most common endocrine disorder, affecting up to 8–13% of women of reproductive age [8]. Key features include infrequent menstrual periods, infertility, hyperandrogenism and insulin resistance. Women with PCOS are at higher risk of developing pregnancy-induced complications [9] and various meta-analyses have shown that the risk of developing pregnancy-induced hypertension and PE in women with PCOS is 3–4 times more than controls [10]. This higher risk of PE in PCOS appears to be independent of age, body mass index (BMI), and nulliparity [11]. Unfortunately, the molecular mechanisms predisposing to increased PE risk in PCOS are unclear [12]. 

This work has studied pre-eclampsia by examining data from molecular up to population scales. It points to a shortage of Coenzyme A (CoA) as a feature leading to the production of signalling lipids and toxic metabolites that induce vasoconstriction, inflammation, oxidative stress, thrombogenesis, acidosis, apoptosis and neurotoxicity. It explains why PCOS is a risk factor for PE and points to potential treatments plus areas for further investigation.

## 2. Results

### 2.1. Metabolomic Data Indicate Restricted Coenzyme A Levels and Signalling Lipid Formation

Four relevant published metabolomics studies in women with pre-eclampsia (aimed at identifying biomarkers) have been combined and summarised in Table S1. While the NMR study identified 12 polar/charged metabolites (including carnitine, 2 aliphatic amino acids and 4 other carboxylic acids), the untargeted mass spectrometry study had 34 significantly changed molecules of which 31 correspond to mostly fatty acyl derivatives (carboxylic acids, carnitines, phosphatidyls and ceramides) or aliphatic amino acid degradation products. Of the latter study, 14 were either signalling molecules (for example, sphingosine-1-phosphate) or markers/agents of adverse physiological conditions found in pre-eclampsia, such as 2-hydroxybutanoate and oxo-methylbutanoic acid (Table 1). When the lipid set was used as a query to determine which enzymes had altered activities (Figure S1), predominantly reactions generating CoA were elevated (namely acyl-CoA thioesterases (ACOT2), carnitine phosphotransferases (CPT1), and 3-hydroxymethyl-3-methylglutaryl-CoA lyase) and reactions consuming CoA were diminished.

### 2.2. Transcriptomic Data in Metabolic Context Can Account for Reduction in Coenzyme A and Observed Lipid Perturbations

The main allosteric regulator of CoA synthesis is pantothenate kinase 1 (PANK1), whose mRNA level in the placenta is usually low (Figure S2), potentially limiting the capacity to produce CoA. Transcriptome analysis [5] reveals that PANK1 mRNA levels are even lower in pre-eclampsia, implying a potential shortage of CoA under these circumstances. This is reinforced by lower mRNA for phosphopantothenoylcysteine decarboxylase, another enzyme in CoA synthesis (Figures S3 and S4). Furthermore, in PE, mRNA levels for cytosolic enzymes generating CoA from acyl derivatives were increased while those consuming it were decreased (Table S2). Transcripts for 15 CoA-producing enzymes were decreased, but these concern detoxification, formation of glycerides or esters, Transcripts for 5 CoA-consuming enzymes were elevated, but their activity is confined to mitochondria, peroxisomes or endoplasmic reticulum. There are two cases where there are elevated transcripts for CoA-consuming and producing enzymes that are adjacent in metabolic pathways, making them effectively CoA neutral (Table S2).

### 2.3. CoA Release and Changes to mRNA Levels Explain Elevated Signalling Lipids and Toxic Compounds

The Brew et al. data [5] show that there are a very large number of transcript-level changes in the PE placenta, many of which encode enzymes or their regulatory subunits and affect many areas of metabolism. Supplementary Figures S5–S13 contain the human KEGG metabolic pathway charts for central carbon metabolism, including aspects of lipid metabolism, and branched-chain amino acid degradation. Enzymes with altered gene expression in PE have been colour coded as described in Figure S3, and the subcellular compartments in which the enzymes reside are provided in Supplementary Table S3. 

**Figure 1:**
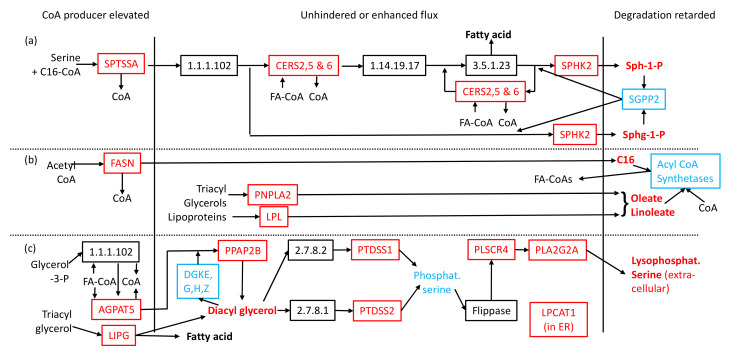
Common metabolic pathway motif. Pathways represent synthesis of (**a**) sphingosine-1-P and sphinganine-1-P, (**b**) oleic and linoleic acids, (**c**) diacyl glycerol and lysophosphatidyl serine. Rectangles represent enzymes present in human metabolism. Enzyme genes with altered transcript levels are named. Metabolites are in unboxed text, with signaling molecules in bold font. Red and blue colouring respectively denote elevated and reduced levels. Arrows denote products/substrates linking enzymes, except at the start/end of pathways. Enzymes degrading lysophosphatidyl serine (LPCATs) are generally confined to the endoplasmic reticulum. Abbreviations: C16—hexadecanoic acid, CoA—Coenzyme A, FA—fatty acyl, P—phosphate, Sph-1-P—sphingosine-1-phosphate, Sphg-1-P—sphinganine-1-phosphate.

Figures S5–S9 reveal a common three-part motif in the synthesis pathways of 8 of the above signalling lipids (see Figure 1): sphinganine-1-phosphate, sphingosine-1-phosphate, linoleic acid, oleic acid, hexadecanoic acid, diacyl glycerols and lysophosphatidyl serine. The first part shows that CoA-releasing enzymes are located early in pathways and have elevated mRNA levels. Purely by mass-action kinetics, low CoA levels will increase flux into these pathways. The second is that the remainder of the pathways contain unchanged or elevated transcript levels of the enzymes involved, meaning that flux to the signalling lipids is unhindered. The third part of the motif consists of lower mRNA levels for enzymes involved in signalling-lipid removal, thereby contributing to the observed elevated levels. A similar motif is seen in ketone body formation (Figure S9), with downregulation of BDH1 suggesting that the observed elevated level of 3-hydroxybutanoic acid may have a substantial component of the toxic valine-degradation product.

Degradation of isoleucine, leucine and valine usually takes place exclusively in mitochondria, but in the placenta the first step, catalysed by a branched-chain aminotransferase (BCAT1), also resides in the cytosol. Transcripts for this enzyme are elevated in PE (Tables S3 and S4, Figure S13), along with many others in the degradation pathway. The next degradative step occurs in mitochondria and requires CoA as a substrate. A shortage of that would reduce flux through the enzyme (branched-chain keto-acid dehydrogenase, BCKDH), leading to a higher concentration of the keto-acid.

### 2.4. Multiple Conflicting Regulatory Pathways Arise from the Elevated Signalling Lipids

PE results in elevated placental transcription of the sphingosine-1-phosphate receptor 3 (S1PR3) gene (Table S4). Via this receptor, sphingosine-1-phosphate appears to be promoting a cytoprotective response, as evidenced by the elevated expression of sterol-regulated binding protein 1c (SREB-1c) and CREB-binding protein (CREBBP). These activate the gene for fatty acid synthase (FASN), which is driven by releasing CoA from acetyl-CoA to form hexadecanoic acid. It is regulated both allosterically and through phosphorylation by AMP-activated protein kinase (PRKAA2). Citrate is increased while hexadecanoyl-CoA inhibits activity, but a shortage of CoA and downregulation of acyl-CoA synthetases can explain the observed elevated levels of this fatty acid. Acyl groups are, however, being shunted, again by mass-action kinetics, from CoA to carnitine, as shown by their observed elevated levels (Table S1). PRKAA2 and its upstream regulator, cAMP-dependent protein kinase (PRKACA,B,G), all have reduced mRNA levels in PE, suggesting that hexadecanoic acid formation is occurring at a high rate.

Hexadecanoic acid will compete with other fatty acids for CoA. This probably accounts for the elevated levels of other long-chain fatty acids, notably linoleic and oleic acids, which are strong activating ligands of peroxisome proliferator-activated receptor alpha (PPARa), whose pathway has already been shown to be active (Table S4). Along with PPAR gamma coactivator-related protein 1 (PPRC1) and Lipin 1 (LPIN1), it stimulates fatty acid degradation by elevating the expression of genes for mitochondrial and peroxisomal beta-oxidation, phosphatidyl-choline transfer protein (PCTP) and for the formation of carnitine and CoA. PCTP forms a complex with mitochondria-associated ACSL4 (which also has elevated mRNAs) to enable free fatty acids to enter mitochondria. The demand for CoA to oxidize longer chain fatty acyls can be mitigated by the observed shunting of some of the lower length fatty acyls (C2, C8, C10 and C12) from CoA to carnitine (Table S1). Thus, the PE placenta has a futile cycle in which fatty acids are being synthesized in the cytosol while being degraded in the organelles. 

Sphingosine-1-phosphate also causes vaso-constriction, which has led to hypoxia, as evidenced by the elevated transcription of the vascular endothelial growth factor B gene (VEGFB) amongst others. This adds to both mitochondrial stress and the cytoprotective response, which creates a positive feedback loop that is liable to progressively elevate levels of shingosine-1-phosphate and other signalling and toxic molecules. Vaso-constriction also leads to turbulent blood flow that is thought to damage syncytiotrophoblasts [1], causing elevated levels of intracellular protein and amino acids in the bloodstream. These stresses have activated programmed cell death (apoptosis), of which some components are elevated in PE, for example, phospholipid scramblase 4, which allows mixing of lipids from one side of the plasma membrane to another.

### 2.5. Population Variation

Population differences may lead to one aspect of the above adverse medical conditions appearing more than another. Genome-wide association studies might shed light on this. A foetal variant is known to interfere with angiogenesis, leading to hypoxia [21]. Maternal GWAS studies [22,23] have been less clear cut. This might not be surprising, given that pre-eclampsia appears to arise from competition between cellular processes rather than a dysregulation of a single regulatory pathway. However, a closer examination of the data may suggest involvement in other relevant (dys)regulatory activity.

Genes identified in this way might regulate the activity of relevant proteins by post-transcriptional and post-translational modification, and intronic and intergenic markers might lie on gene-regulatory regions that are hundreds of kilobases away from their target gene(s). In this regard, the Zhao et al. data [23] (Table S5) reveal 2 non-coding RNAs and 5 transcription factors of which one (FGF14) is downregulated in PE, a second (ESRRG) might interfere with oestrogen regulation, and a third (RUNX1) regulates PCTP [24] and is found in transcriptional complexes with CREBBP and HIF1A—the main hypoxia sensor (Table S5). Furthermore, there are 4 loci in the upregulated MYC-binding protein 2 gene (MYCBP2), which encodes an enzyme that targets its substrate proteins for degradation. 

The earlier GWAS study [22] also identified a marker near the MYCBP2 gene (Table S6), plus the upregulated RAB10 transcription factor and CHMP2B vesicle-sorting protein, and a non-coding RNA. This study paid particular attention to a highly scoring locus, near which are a transcription factor required for suppression of apoptosis (RALB) and a downregulated non-coding RNA (LOC84931, which is associated with poor survival from cancer). There are several up-regulated genes of interest close to the loci in both studies. From Table S5, there are BCAT1 and lipoprotein lipase (LPL), while from Table S6 there is hydroxyacyl-CoA dehydrogenase alpha (HADHA, involved in beta-oxidation).

Within the population, it is possible that some patients only exhibit particular aspects of PE, for which the above observations may provide an explanation of the root cause. For example, hypertension and the inflammatory response may have their origins in elevation of sphingosine-1-phosphate. In the absence of hypoxia, mitochondrial stress can lead to acidosis from elevated lactate and other hydroxyacids. Moreover, thrombosis arises for a variety of reasons, but a contributing factor could be placental oxidative stress triggering apoptosis. Finally, early elevated expression of PCTP and/or a high leucine concentration could contribute to insulin resistance.

## 3. Discussion

### 3.1. Systems Pathology in PE

In PE, there is a conflict of interest between the normal maternal physiology, namely programmed cell death in the face of hypoxia and mitochondrial stress, and the foetus, which needs a viable placenta for survival. This conflict leads to interference in the various regulatory processes involved, which lead to the build-up of toxic metabolites and adverse antenatal conditions. At least six examples of interference are present.

First, CoA synthesis is stimulated by PPARa [25] to allow efficient fatty acid oxidation, but all the above observations indicate that in PE it is significantly inhibited (Figure S14). Second, in a normal pregnancy, the placenta has elevated mRNA levels of the oestrogen receptor. Upon binding the hormone, the receptor inhibits PPARa activity [26] so that more fatty acids are available for the foetus. However, in PE, the mRNA level is not high, thereby potentially reducing inhibition of PPARa (Figure S14). Likewise, third, insulin inhibits PPARa activity for similar reasons via the mTORC1 pathway [27], but in PE, even though transcription of the insulin receptor is elevated, the inhibitory effect is blocked by the elevated levels of the ACOT13–PCTP complex [28] and leucine [29] (Figure S15). The effect of the latter on other tissues can lead to insulin resistance, the observed elevated plasma glucose in PE and perhaps gestational diabetes.

The fourth example is the dysregulation of leucine/isoleucine/valine degradation in mitochondria (Figure S16). The products of the elevated amino acid transaminase (BCAT1,2) activity enter the branched-chain keto-acid dehydrogenase complex (BCKDH) to combine with CoA forming keto-acyl CoAs. BCKDH is allosterically inhibited by high NADH and inactivated by branched-chain keto acid dehydrogenase kinase (BCKDK). The latter has a higher level of mRNA in PE but is also allosterically inhibited by elevated levels of keto-acids [30]. It remains unclear how much BCKDH activity is affected, as low CoA levels can explain the elevated level of isoketovalerate, which may be inhibiting the kinase activity. Some BCKDH activity does remain; however, because other degradation intermediates are observed (Figure S13), of which 3-hydroxybutyrate and 3-hydroxyvalerate are known to cause mitochondrial stress.

The fifth example concerns the metabolism of phosphatidyl serines (Figure S17). Under normal conditions, these are synthesised in the internal membrane surface of the endoplasmic reticulum and then transferred back to the cytosolic face by flippases in the Golgi [31]. In PE, their synthesis is likely to be elevated as both the precursors, diacyl glycerols, and mRNAs for enzymes catalysing their production are raised. However, mitochondrial/apoptotic stress has triggered the expression of phospholipid scramblase 4 (PLSCR4) and anoctamins (ANO2, 6 and 10). These flip the phosphatidyl serines back to the outer surface of the plasma membrane, where an extracellular phospholipase A2 (PLA2G2A) catalyses an overall depletion of phosphatidyl serine to produce an excess of lysophosphatidyl serine. 

The final example shows that dysregulation is occurring at the heart of carbon metabolism (Figure S18). Pyruvate dehydrogenase (PDH) links glycolysis to acetyl CoA and the Krebs cycle and is tightly regulated by inhibitory protein kinases. Under normal circumstances and for different reasons, hypoxia and oxidative stress increase the transcription of the gene for PDH Kinase-1 [32], while insulin represses the expression of the PDH kinase-4 gene [33]. In PE, these are not happening and mRNA for PDH kinase-1 is reduced. PPARa and hypoxia are known to elevate PDH Kinase-4 (PDK4) gene transcription [34,35]. This, along with the degradation of certain amino acids, can account for the observed raised levels of pyruvate and lactate in PE. 

**Figure 2:**
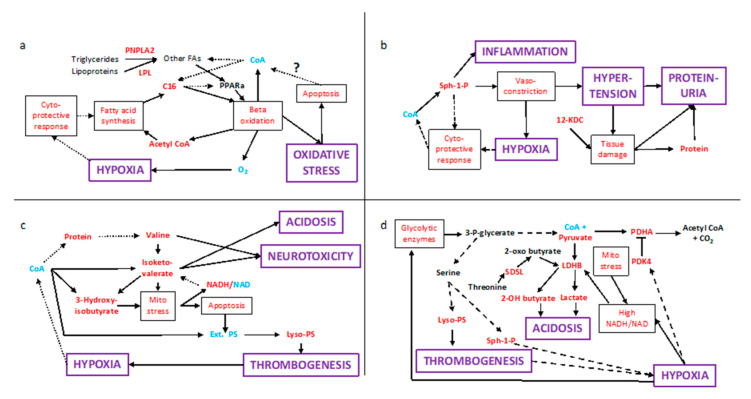
Clinical signs linked to CoA restriction. (**a**) futile cycle linking to hypoxia and oxidative stress; (**b**) Sphingosine-1-phosphate cycle linked to hypoxia, hypertension and proteinuria; (**c**) perturbed valine metabolism effecting thrombogenesis, acidosis and neurotoxicity; (**d**) central carbon metabolism links acidosis with other conditions. Purple and black boxes respectively represent clinical signs and physiological/biochemical processes, with unboxed nodes corresponding to molecules. Red, blue and black text respectively refer to higher, lower and unchanged activity or molecular concentration. Solid and dashed arrows denote direct and indirect effects or interactions. The barred arrow between PDK4 and PDHA indicates an inhibition event. Abbreviations: 12-KDC—12-Ketodeoxycholate, but—butyric acid, C16—palmitic acid, CoA—Coenzyme A, Ext. PS—External phosphatidylserine, Fas—Fatty acids, LDHB—Lactate Dehydrogenase B, LPL—Lipoprotein Lipase, Lyso-PS—Lysophosphatidyl serine, Mito—mitochondrial, NADH/NAD—ratio of NADH to NAD, PDHA—Pyruvate Dehydrogenase A, PDK4—Pyruvate Dehydrogenase Kinase 4, PNPLA2—Potatin-like phospholipase domain containing 2, PPARa—Peroxisome proliferator-activated receptor alpha, SDSL—Serine/Threonine Dehydratase-like, Sph-1-P—Shingosine-1-phosphate.

Once regulatory pathways are considered, the above transcriptional changes create interlinked positive feedback loops, which appear to cause the levels of signalling and toxic molecules to increase over time, leading to adverse medical conditions. The futile cycle involving synthesis and degradation of fatty acids causes localised hypoxia, and hence mitochondrial stress and apoptosis (Figure 2a). Figure 2b shows a double feedback loop involving sphingosine-1-phosphate, in which high levels cause an inflammatory response and vasoconstriction leading to hypertension and proteinuria. The latter may be exacerbated by the observed raised levels of ketodeoxycholate (12-KDC), which disrupts membranes, potentially causing further damage to the placenta, but also to kidneys, other organs and contributing to haemolysis. 

Likewise, there is a double feedback loop involving valine and isoketovalerate (Figure 2c). The latter causes mitochondrial stress [36], elevating NADH, which is an allosteric inhibitor of BCKDH and resulting in less isoketovalerate being esterified to CoA. Elevation of isoketovalerate is exacerbated by the placenta having high levels of both cytosolic BCAT1 and valine. In PE patients, the elevated levels of valine and its ketoacid become an issue for other tissues with high BCAT1 levels, notably the pancreas, brain, endometrium and ovaries (Figure S22). This provides an explanation for the neurotoxicity associated with PE, and acidosis may also explain liver failure in PE patients. On rare occasions, PE also affects the pancreas [37,38]. There is a second loop linking apoptosis, thrombosis and hypoxia. The release of microvesicles [1] rich in lysophosphatidyl serine could be a factor causing disseminated intravascular coagulation.

Figure 2d shows how the various factors influence the heart of carbon metabolism. Hypoxia elevates expression of the inhibitory PDK4 and acts on two other fronts. It elevates glycolysis resulting in elevated pyruvate levels; glucogenic amino acid degradation might contribute to this. A glycolytic intermediate is the substrate of phosphoglycerate dehydrogenase which is the first step to serine synthesis, and its elevated gene expression (Table S4) can ensure a supply to the amino acid to form sphingosine-1-phosphate and lysophosphatidyl serine. Finally, a high NADH/NAD ratio favours the reactions from pyruvate and a threonine degradation product to the respective acidogens lactate and 2-hydroxybutyrate, which is also true of ketoacids in branched-chain amino acid degradation.

**Figure 3:**
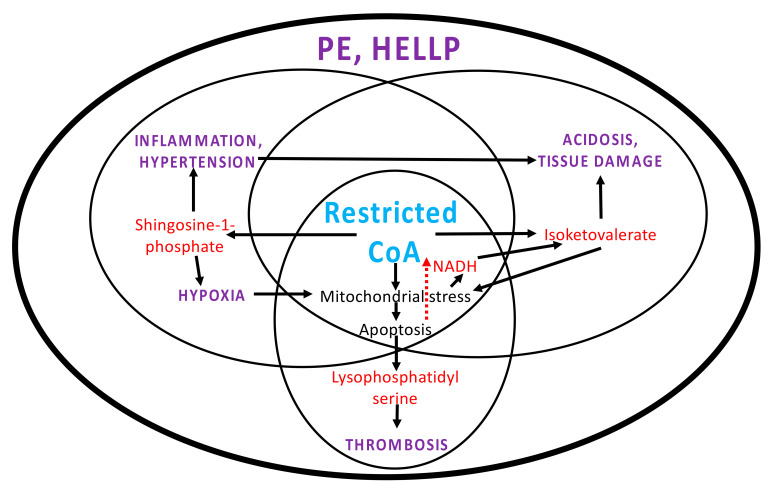
Interlinking of separate adverse conditions. Separate adverse cycles could be causing different adverse conditions. However, these come together in PE and the perfect storm of HELLP. Arrows denote links between metabolites, cellular states and disease conditions. The potential link between apoptosis and restricted CoA is represented by a dashed red arrow.

Given the separate feedback loops involving sphingosine-1-phosphate, isoketovalerate and lysophosphatidyl serine (apoptosis), one can view this as three separate adverse conditions respectively leading to hypertension and/or inflammation, acidosis and thrombosis. These separate cycles are linked, however, with one potentially causing others to occur (Figure 3). From this perspective, pre-eclampsia is the result of all three occurring together and a ‘perfect storm’ in HELLP syndrome, where there is membrane disruption and platelet depletion as well. This perspective could explain why separate GWAS studies have not been consistent, because multiple adverse conditions are involved.

### 3.2. PCOS and Other Risk Factors

PCOS, diabetes and insulin resistance are known risk factors for PE, and the above observations provide an explanation for this. They all have high blood triglycerides, which, after entering syncytiotrophoblasts, release fatty acids. These activate PPARa because they are not sufficiently converted to CoA derivatives. Furthermore, PE induces an increase in levels of a triglyceride lipase (PNPLA2) and LPL which increase the rate of formation of cytosolic fatty acids (Figure 2a). A previous history of PE is also a risk factor, which can be explained by the elevated blood valine and isoketovalerate levels damaging the endometrium and ovaries owing to their high levels of BCAT1 and other tissues through oxidative stress inducing DNA damage [39]. The latter, along with hypertension and thrombogenesis, may also explain why women with a history of PE have double the risk of early cardiac, cerebrovascular, and peripheral arterial disease, and cardiovascular mortality.

### 3.3. Apoptosis and CoA

Normal pregnancy requires programmed cell death at several stages to ensure healthy development, but in PE this process has gone awry [40]. There is a long track record of Vitamin B5, the precursor of CoA, reducing disease severity by protecting tissues against the effects of apoptosis [41]. However, a big issue for PE is why does PANK1 mRNA, and hence enzyme, levels drop? It has been shown in yeast that apoptosis itself represses this gene [42]. It is logical for a process causing cell death to lower levels of CoA, which lies at the heart of energy production, lipid metabolism and membrane synthesis. Perhaps, the same is happening here (Figure 2a). Certainly, Vitamin B5 and apoptosis oppose each other—the former for ‘cell health’, the latter for cell death.

### 3.4. Potential Avenues for Translation, Prevention and Treatment

Low CoA levels appear to be a root cause of PE and may contribute to hypertension and thrombosis. Translating these findings into clinical practice requires further studies beyond the scope of this work. While direct CoA measurement in first-trimester placenta would be ideal, the current assays only detect free CoA or its acetyl derivative, for example, Shurubor et al. (2017) [43], but do not detect longer length acyl derivatives. Moreover, testing once symptoms of pre-eclampsia appear would be late, because the catastrophic effects of CoA restriction are already well underway. 

It is, however, more important to start investigating levels of blood sphingosine-1-phosphate, lysophosphatidyl serines, branched-chain amino acids and isoketovalerate. Ideally, an assay, analogous to the point of care tests for blood glucose, where only a spot of blood is required, and results come back in under a minute. This could then be rolled out so mothers can be tested when they first know they are pregnant and then again after 12–14 weeks. If levels are already high or are noticeably worse by the second test, then more preventative action can be taken. This work provides a biochemical basis for recommending a low-fat diet, to reduce the risk of PPARa activation, and moderate exercise, to improve the oxygen supply and reduce the risk of hypoxia. 

Vitamin B5 supplementation might improve CoA levels, stalling the onset of PPARa activation. A clinical trial to see if this significantly reduces pre-eclampsia and other adverse antenatal conditions is not unreasonable, especially as a 2009 Danish study found that regular periconceptional multivitamin (including vitamin B5) use was associated with a reduced risk of pre-eclampsia [44]. Increasing PANK1 activity might also help, either by altering its allostery (for example, with pantazines [45]) or affecting upstream genetic regulators to increase transcription.

Preventative treatment approaches that target the gut microbiome are another avenue to explore. Gut microbiota are an important source of Vitamin B5 [46]. Recent research suggests that disruptions in the composition of the gut microbiome may play a role in the pathogenesis of PE [47]. The Norwegian Mother and Child Cohort Study demonstrated that probiotic milk intake lowered the risk of developing pre-eclampsia, an effect not observed when the probiotic was taken before or early in pregnancy [47]. Dietary counselling to encourage high fibre intake in pregnant women should be assessed further as a potential preventative and management strategy in pre-eclampsia [48], as it may optimise Vitamin B5 and CoA biosynthesis by gut microbiota. 

Screening at 14–16 weeks for elevated sphingosine-1-phosphate and isoketovalerate are probably early risk indicators of hypertension, acidosis and PE, as they imply that the placenta is already stressed. At this point, or once symptoms have appeared, then other treatments may be effective. Reducing sphingosine-1-phosphate binding to the receptors causing vasoconstriction is an obvious target for reducing hypertension and perhaps hypoxia. However, selective inhibition PPARa activity could also reap benefits. In this regard, blocking the inhibitory effect of PCTP/ACOT13 and leucine might enable insulin to suppress PPARa activity for longer and avoid insulin resistance. There is also a case for further investigation of lysophosphatidyl serine to counteract its effects on thrombogenesis, which might be assisted by reducing acidosis. 

## 4. Data and Methods

The metabolomics datasets used in this study are Kenny et al. 2010 [49], Bahado-Singh et al. 2017 [50], Odibo et al. 2011 [51] and Dunn et al. 2009 [52]. Protein subcellular localisation data were obtained from The Uniprot Consortium [53]. Robust transcriptomic data were obtained from a systematic study by Brew et al. 2016 [5]. GWAS data were obtained from Johnson et al. 2012 [22] and Zhao et al. 2013 [23]. Genes in the vicinity of significant GWAS loci were identified using the EnsEMBL genome browser (https://www.ensembl.org/, accessed 8 November 2021). Human metabolic pathway charts were downloaded from KEGG [54], and tissue-expression profiling data were obtained from the NCBI (https://www.ncbi.nlm.nih.gov/gene/, accessed 4 November 2021). Lipid information was obtained from HMDB [13] and LipidMaps (https://www.lipidmaps.org, accessed 6 November 2021). 

All figures were produced in Microsoft PowerPoint and converted to PDF files where necessary. All tables were produced in Microsoft Excel.

## 5. Conclusions

This work clearly points to CoA restriction as a major factor underpinning PE and related antenatal conditions, while also providing an explanation for why PCOS and other conditions are risk factors. These observations point to further avenues of clinical research, which may well lead to major improvements in antenatal treatment. Given the central role played by CoA, its restriction may also underpin other complex disease conditions.

## Figures and Tables

**Table 1:** Elevated bioactive lipids and markers.

Molecule. ** Denotes Membership of the Kenny PE-Predictive Set (14–16 wks.)	Comment	Human Metabolome DataBase ID [13]/LipidMaps ID
Linoleic acid Oleic acid ** Hexadecanoic acid	Linoleate and oleate are activating ligands of peroxisome-proliferator activating receptor-alpha (PPARa), which stimulates peroxisome formation and beta-oxidation [14]	HMDB0000673 HMDB0000207 HMDB0000220
Sphingosine-1-phosphate ** Sphinganine-1-phosphate **	Through their receptors mediate a wide range of responses including vasoconstriction, inflammation and cytoprotection [15]	HMDB0000277 HMDB0001383
Eicosatrienoic acid	Can be synthesised in the endoplasmic reticulum by fatty-acyl elongation. Competes with arachidonic acid for cytochrome oxidase and lipoxygenase activities.	HMDB0002925
Octadecenoyl-sn-glycero-3-phosphoserine Dioctanoyl-sn-glycero-3-phosphocholine	Lysophosphatidyl serines and phosphatidyl cholines promote thrombogenesis in the context of lipid bilayers [16]	HMDB0240603 LMGP01011251
Hexadecenoyl-eicosatetraenoyl-sn-glycerol ** Di-(octadecadienoyl_sn-glycerol **	Diacyl glycerols allosterically activate Protein Kinase C, exerting diverse physiological effects	HMDB0007141 LMGL02010063
12-Ketodeoxycholic acid	Ketodeoxycholates are membrane disruptors. Elevated levels presumably due to dysregulation by liver [17]	HMDB0000328
Oxo-methyl butanoic acid **	Valine degradation product also known as isoketovaleric acid: Neurotoxic, acidogen and metabotoxic	HMDB0000019
3-hydroxybutanoic acid	Comprises 2 isomers. One is a ketone body. The other (3-hydroxy-isobutanoic acid) is a valine degradation product causing mitochondrial stress and lactic acidaemia	HMDB0000442 HMDB0000023
3-Hydroxyisovaleric acid	Leucine degradation product; can cause mitochondrial stress, acidosis and metabotoxicity	HMDB0000754
2-hydroxybutanoic acid	Product of threonine degradation and glutathione synthesis. Marker for insulin resistance and acidosis, indicative of high NADH/NAD ratio [18]	HMDB0000008
Gamma-butyrolactone **	Hydrolyses to gamma-hydroxybutyrate (HMDB0000710) which affects the nervous system, and at high concentrations is an acidogen and neurotoxin [19]	HMDB0000549
Oxolan-3-one **	Marker for lactic acid acidosis [20]	HMDB0002523

## Data Availability

The data presented in this study comprise a synthesis and further annotation from cited sources and can be found in Table 1, Supplementary Tables S1–S6, and Supplementary Figures S8–S17.

## Supplementary Materials

The following supporting information can be downloaded at: https://www.mdpi.com/article/10.3390/ijms23052785/s1.
