# Supplementary material for: Coenzyme A Restriction as a Factor Underlying Pre-Eclampsia with Polycystic Ovary Syndrome as a Risk Factor
Source: Int J Mol Sci. 2022 Mar 3;23(5):2785. doi: 10.3390/ijms23052785 (PMC8911031; doi:10.3390/ijms23052785)
Supplement: Supplementary file 1 [file ijms-23-02785-s001.zip › Hodgman-etal-2022-Figures.pptx]

## Slide 1
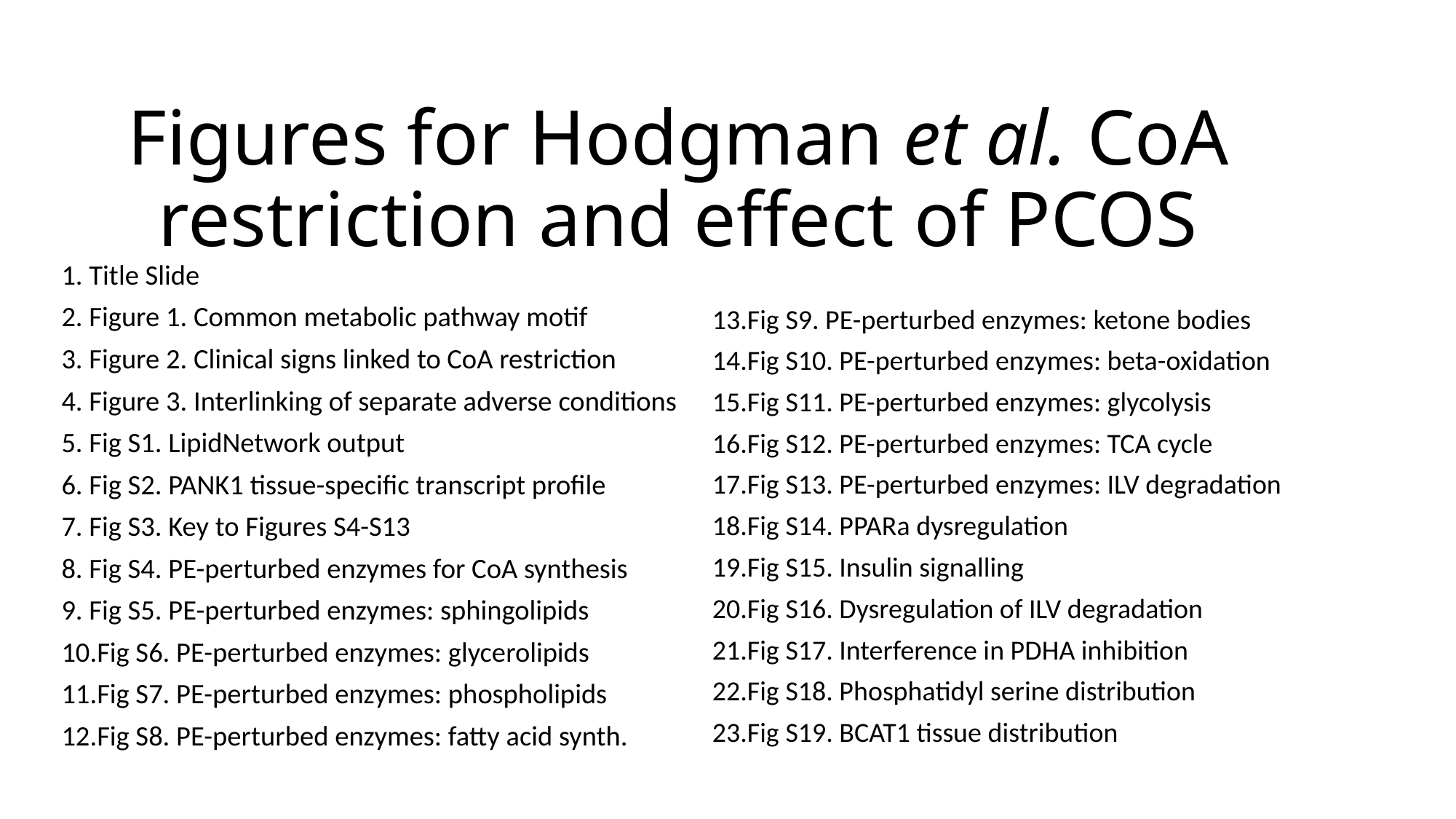

# Figures for Hodgman et al. CoA restriction and effect of PCOS
13.Fig S9. PE-perturbed enzymes: ketone bodies
14.Fig S10. PE-perturbed enzymes: beta-oxidation
15.Fig S11. PE-perturbed enzymes: glycolysis
16.Fig S12. PE-perturbed enzymes: TCA cycle
17.Fig S13. PE-perturbed enzymes: ILV degradation
18.Fig S14. PPARa dysregulation
19.Fig S15. Insulin signalling
20.Fig S16. Dysregulation of ILV degradation
21.Fig S17. Interference in PDHA inhibition
22.Fig S18. Phosphatidyl serine distribution
23.Fig S19. BCAT1 tissue distribution
1. Title Slide
2. Figure 1. Common metabolic pathway motif
3. Figure 2. Clinical signs linked to CoA restriction
4. Figure 3. Interlinking of separate adverse conditions
5. Fig S1. LipidNetwork output
6. Fig S2. PANK1 tissue-specific transcript profile
7. Fig S3. Key to Figures S4-S13
8. Fig S4. PE-perturbed enzymes for CoA synthesis
9. Fig S5. PE-perturbed enzymes: sphingolipids
10.Fig S6. PE-perturbed enzymes: glycerolipids
11.Fig S7. PE-perturbed enzymes: phospholipids
12.Fig S8. PE-perturbed enzymes: fatty acid synth.

## Slide 2
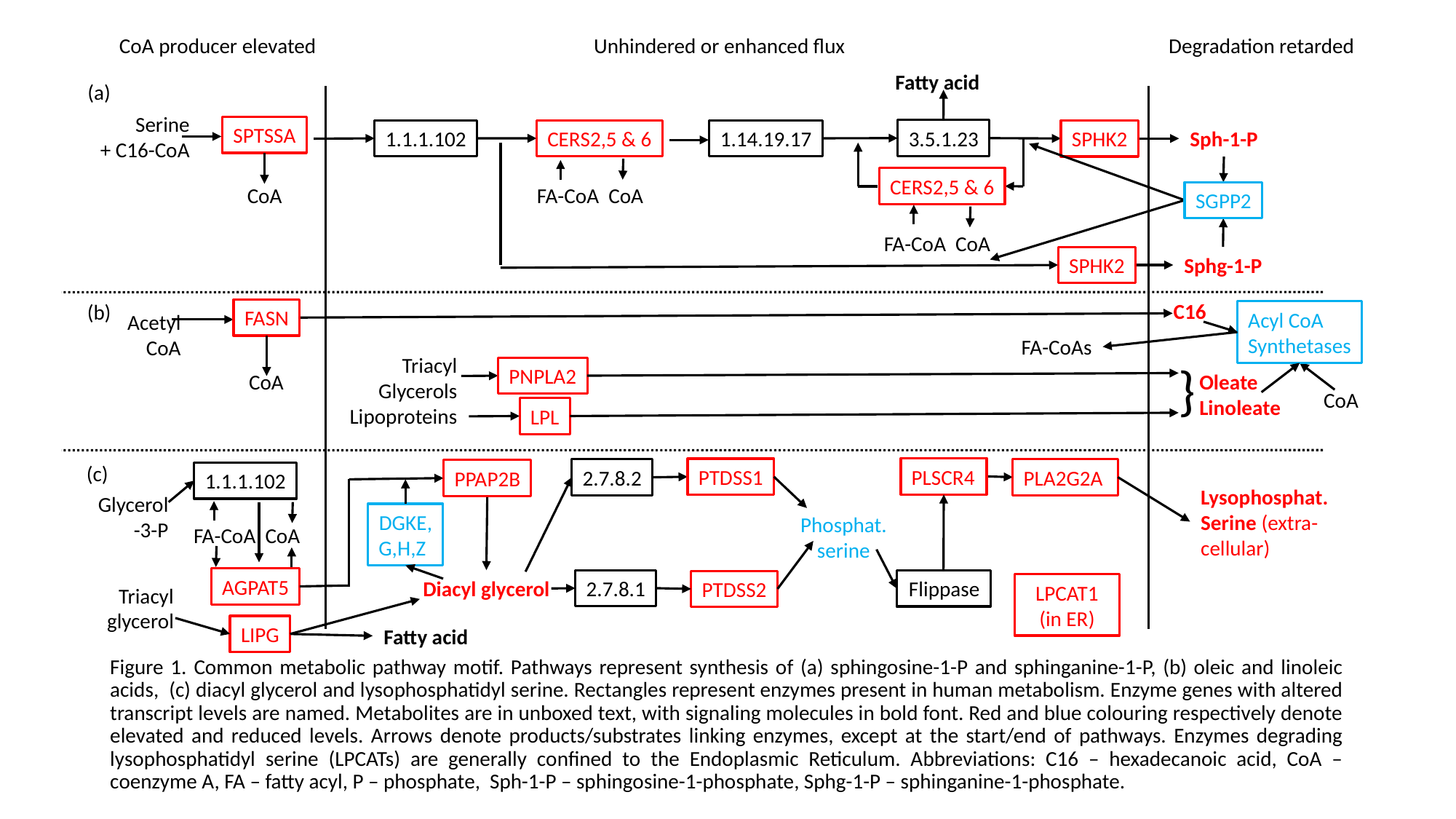

CoA producer elevated
Unhindered or enhanced flux
Degradation retarded
Fatty acid
(a)
Serine
+ C16-CoA
SPTSSA
3.5.1.23
CERS2,5 & 6
1.14.19.17
1.1.1.102
SPHK2
Sph-1-P
CERS2,5 & 6
CoA
FA-CoA CoA
SGPP2
FA-CoA CoA
SPHK2
Sphg-1-P
C16
(b)
FASN
Acyl CoA
Synthetases
Acetyl
CoA
FA-CoAs
Triacyl
Glycerols
Lipoproteins
}
PNPLA2
CoA
Oleate
Linoleate
CoA
LPL
(c)
PLSCR4
PTDSS1
PLA2G2A
2.7.8.2
PPAP2B
1.1.1.102
Lysophosphat.
Serine (extra-
cellular)
Glycerol
-3-P
DGKE,
G,H,Z
Phosphat.
serine
FA-CoA CoA
AGPAT5
2.7.8.1
Diacyl glycerol
Flippase
PTDSS2
LPCAT1
(in ER)
Triacyl
glycerol
LIPG
Fatty acid
Figure 1. Common metabolic pathway motif. Pathways represent synthesis of (a) sphingosine-1-P and sphinganine-1-P, (b) oleic and linoleic acids, (c) diacyl glycerol and lysophosphatidyl serine. Rectangles represent enzymes present in human metabolism. Enzyme genes with altered transcript levels are named. Metabolites are in unboxed text, with signaling molecules in bold font. Red and blue colouring respectively denote elevated and reduced levels. Arrows denote products/substrates linking enzymes, except at the start/end of pathways. Enzymes degrading lysophosphatidyl serine (LPCATs) are generally confined to the Endoplasmic Reticulum. Abbreviations: C16 – hexadecanoic acid, CoA – coenzyme A, FA – fatty acyl, P – phosphate, Sph-1-P – sphingosine-1-phosphate, Sphg-1-P – sphinganine-1-phosphate.

## Slide 3
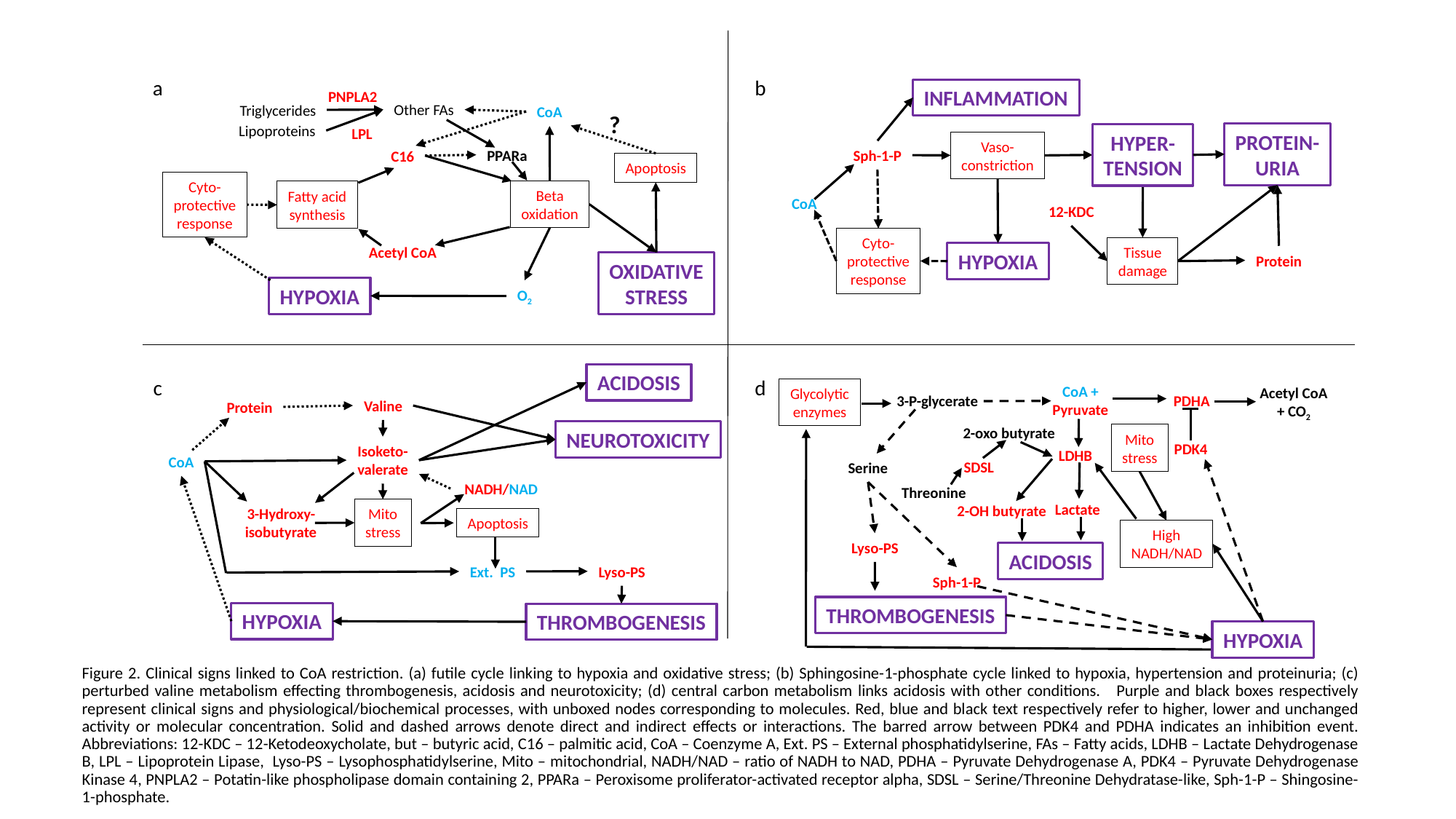

a
b
INFLAMMATION
PNPLA2
Other FAs
Triglycerides
CoA
?
Lipoproteins
LPL
PROTEIN-
URIA
HYPER-
TENSION
Vaso-
constriction
PPARa
Sph-1-P
C16
Apoptosis
Cyto-
protective
response
Beta
oxidation
Fatty acid
synthesis
CoA
12-KDC
Cyto-
protective
response
Acetyl CoA
Tissue
damage
HYPOXIA
Protein
OXIDATIVE
STRESS
HYPOXIA
O2
ACIDOSIS
d
c
CoA +
Pyruvate
Acetyl CoA
+ CO2
Glycolytic
enzymes
PDHA
3-P-glycerate
Valine
Protein
2-oxo butyrate
NEUROTOXICITY
Mito
stress
PDK4
Isoketo-
valerate
LDHB
CoA
SDSL
Serine
NADH/NAD
Threonine
Lactate
2-OH butyrate
3-Hydroxy-
isobutyrate
Mito
stress
Apoptosis
High
NADH/NAD
Lyso-PS
ACIDOSIS
Lyso-PS
Ext. PS
Sph-1-P
THROMBOGENESIS
HYPOXIA
THROMBOGENESIS
HYPOXIA
Figure 2. Clinical signs linked to CoA restriction. (a) futile cycle linking to hypoxia and oxidative stress; (b) Sphingosine-1-phosphate cycle linked to hypoxia, hypertension and proteinuria; (c) perturbed valine metabolism effecting thrombogenesis, acidosis and neurotoxicity; (d) central carbon metabolism links acidosis with other conditions. Purple and black boxes respectively represent clinical signs and physiological/biochemical processes, with unboxed nodes corresponding to molecules. Red, blue and black text respectively refer to higher, lower and unchanged activity or molecular concentration. Solid and dashed arrows denote direct and indirect effects or interactions. The barred arrow between PDK4 and PDHA indicates an inhibition event. Abbreviations: 12-KDC – 12-Ketodeoxycholate, but – butyric acid, C16 – palmitic acid, CoA – Coenzyme A, Ext. PS – External phosphatidylserine, FAs – Fatty acids, LDHB – Lactate Dehydrogenase B, LPL – Lipoprotein Lipase, Lyso-PS – Lysophosphatidylserine, Mito – mitochondrial, NADH/NAD – ratio of NADH to NAD, PDHA – Pyruvate Dehydrogenase A, PDK4 – Pyruvate Dehydrogenase Kinase 4, PNPLA2 – Potatin-like phospholipase domain containing 2, PPARa – Peroxisome proliferator-activated receptor alpha, SDSL – Serine/Threonine Dehydratase-like, Sph-1-P – Shingosine-1-phosphate.

## Slide 4
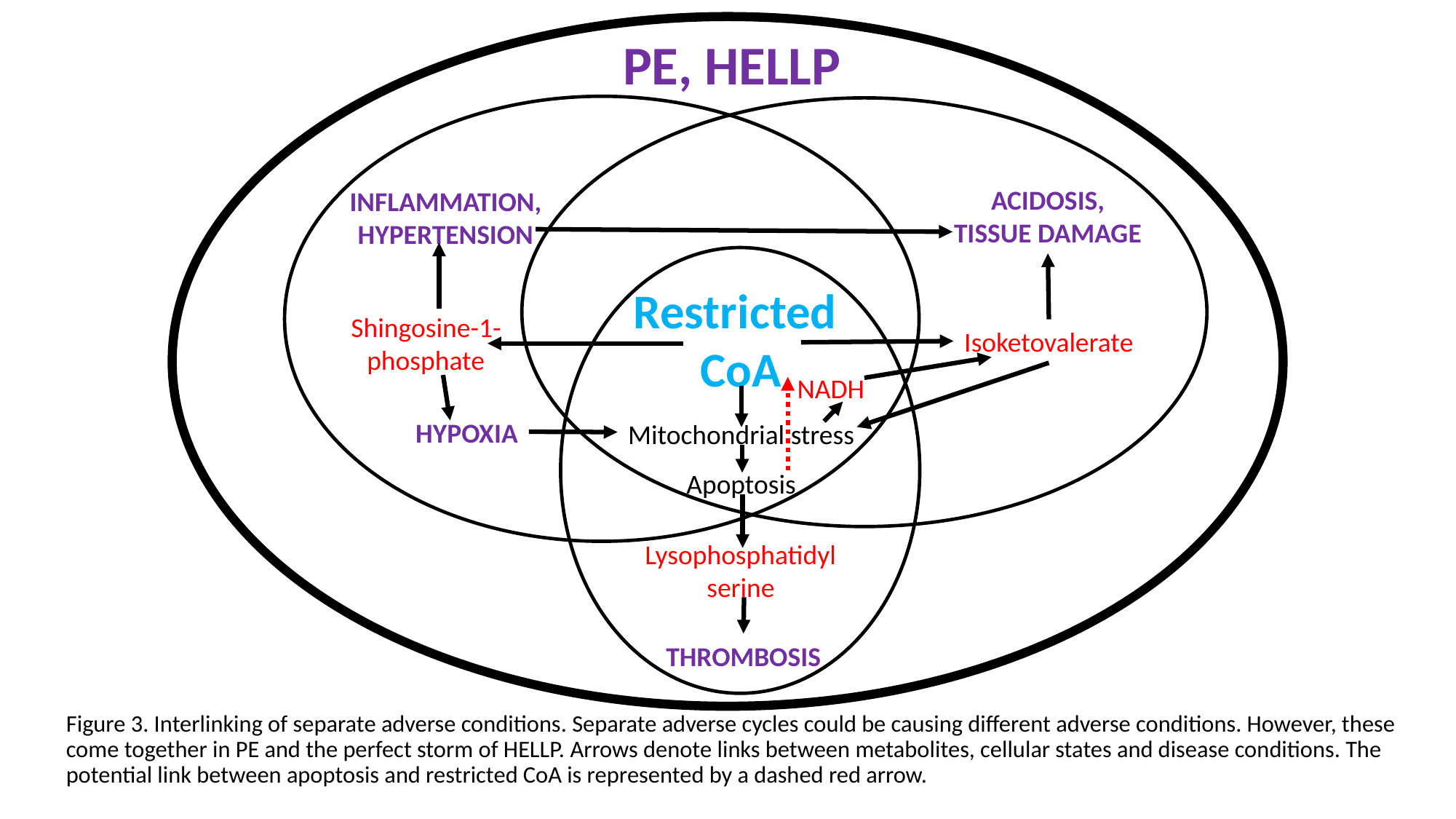

PE, HELLP
ACIDOSIS,
TISSUE DAMAGE
INFLAMMATION,
HYPERTENSION
Restricted
CoA
Shingosine-1-
phosphate
Isoketovalerate
NADH
HYPOXIA
Mitochondrial stress
Apoptosis
Lysophosphatidyl
serine
THROMBOSIS
Figure 3. Interlinking of separate adverse conditions. Separate adverse cycles could be causing different adverse conditions. However, these come together in PE and the perfect storm of HELLP. Arrows denote links between metabolites, cellular states and disease conditions. The potential link between apoptosis and restricted CoA is represented by a dashed red arrow.

## Slide 5
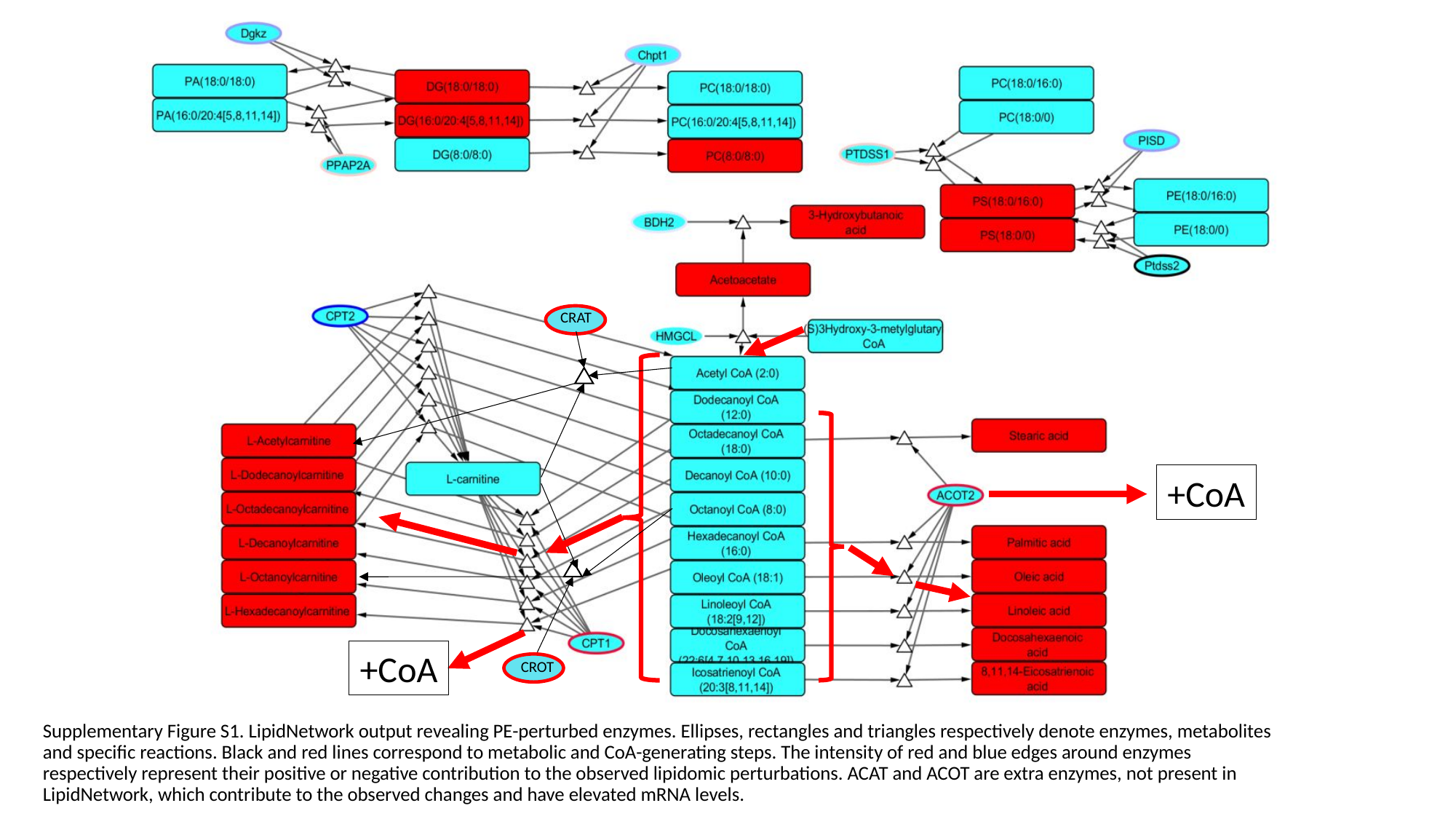

CRAT
+CoA
+CoA
CROT
Supplementary Figure S1. LipidNetwork output revealing PE-perturbed enzymes. Ellipses, rectangles and triangles respectively denote enzymes, metabolites and specific reactions. Black and red lines correspond to metabolic and CoA-generating steps. The intensity of red and blue edges around enzymes respectively represent their positive or negative contribution to the observed lipidomic perturbations. ACAT and ACOT are extra enzymes, not present in LipidNetwork, which contribute to the observed changes and have elevated mRNA levels.

## Slide 6
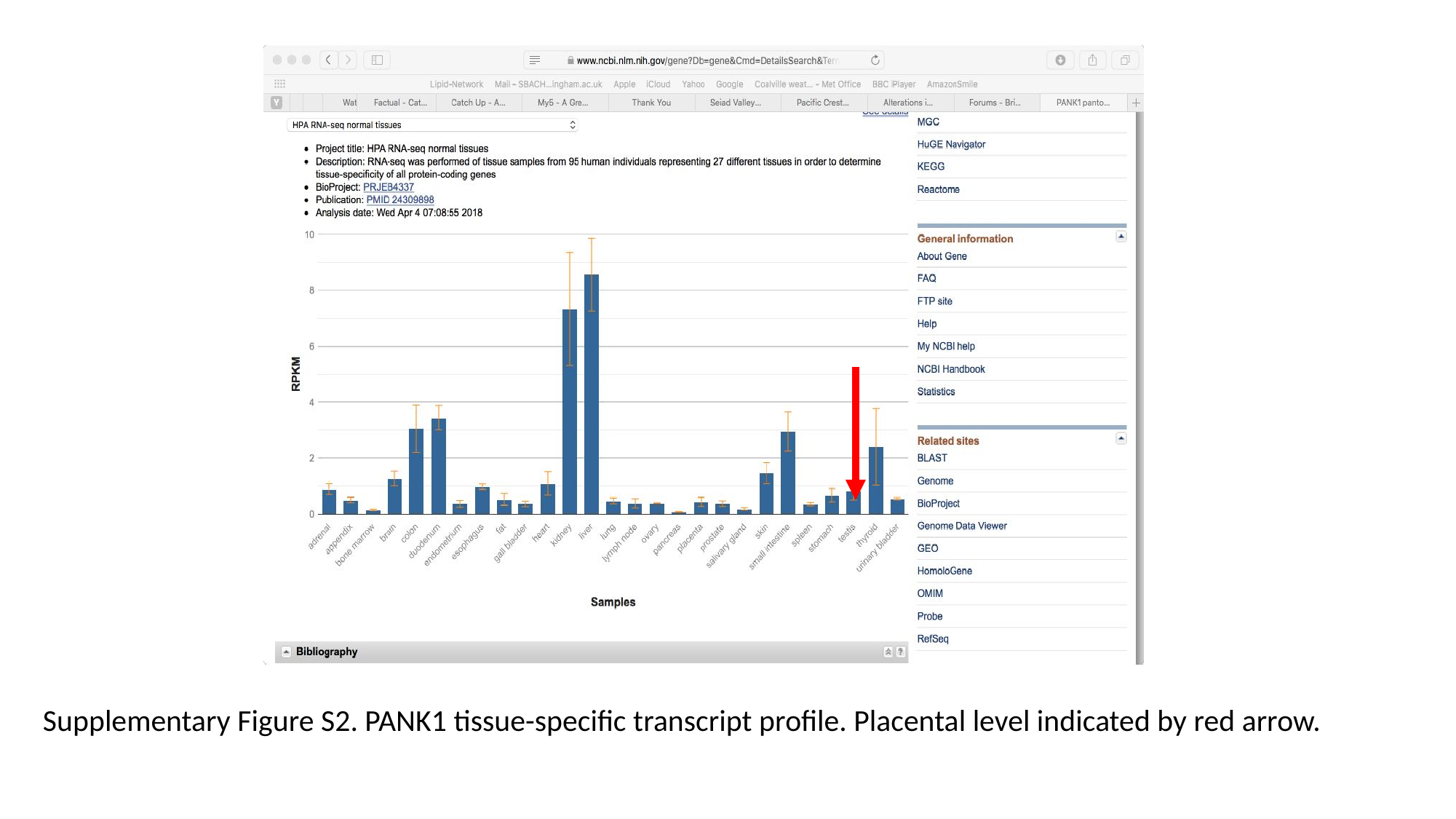

Supplementary Figure S2. PANK1 tissue-specific transcript profile. Placental level indicated by red arrow.

## Slide 7
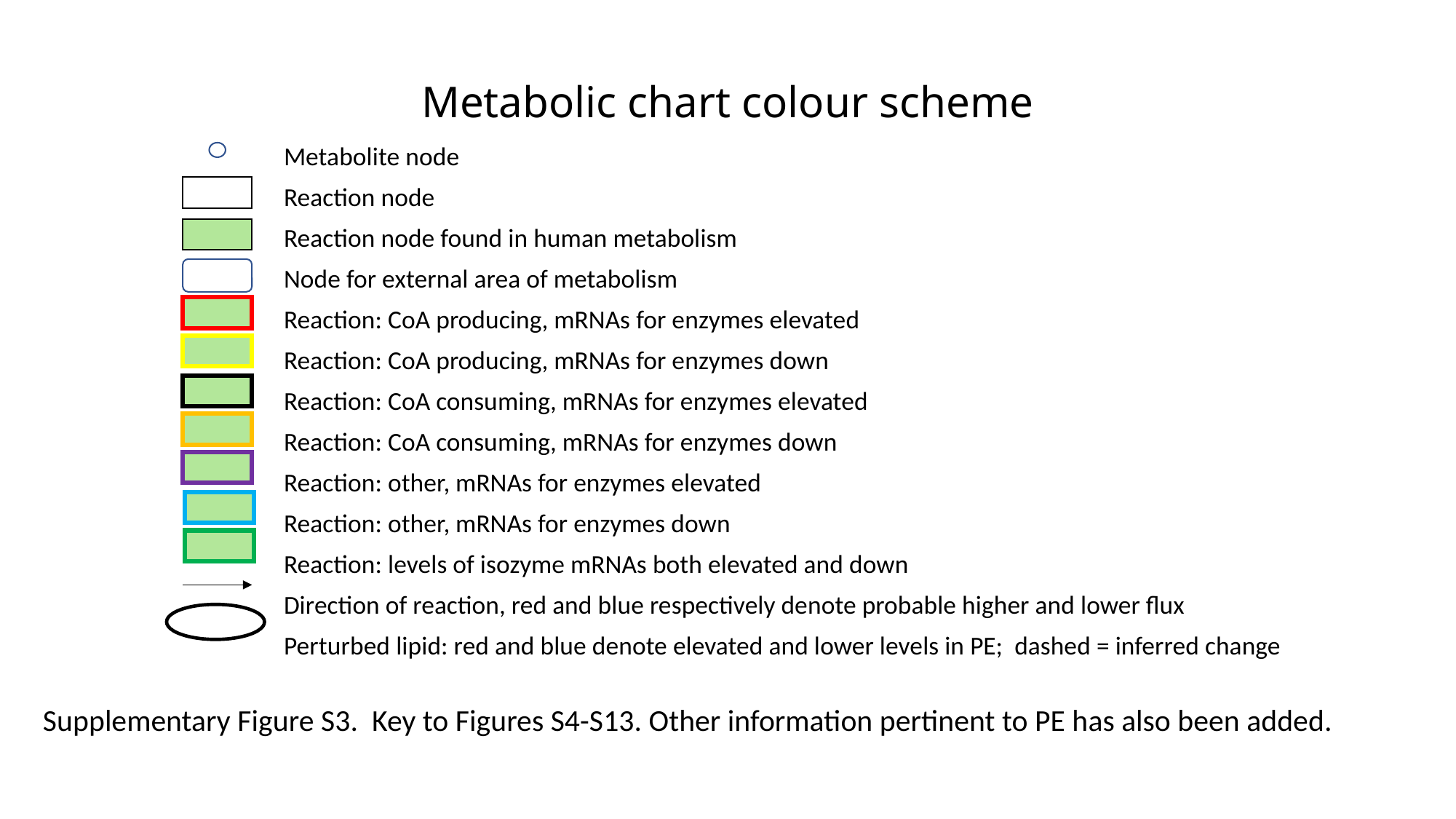

# Metabolic chart colour scheme
Metabolite node
Reaction node
Reaction node found in human metabolism
Node for external area of metabolism
Reaction: CoA producing, mRNAs for enzymes elevated
Reaction: CoA producing, mRNAs for enzymes down
Reaction: CoA consuming, mRNAs for enzymes elevated
Reaction: CoA consuming, mRNAs for enzymes down
Reaction: other, mRNAs for enzymes elevated
Reaction: other, mRNAs for enzymes down
Reaction: levels of isozyme mRNAs both elevated and down
Direction of reaction, red and blue respectively denote probable higher and lower flux
Perturbed lipid: red and blue denote elevated and lower levels in PE; dashed = inferred change
Supplementary Figure S3. Key to Figures S4-S13. Other information pertinent to PE has also been added.

## Slide 8
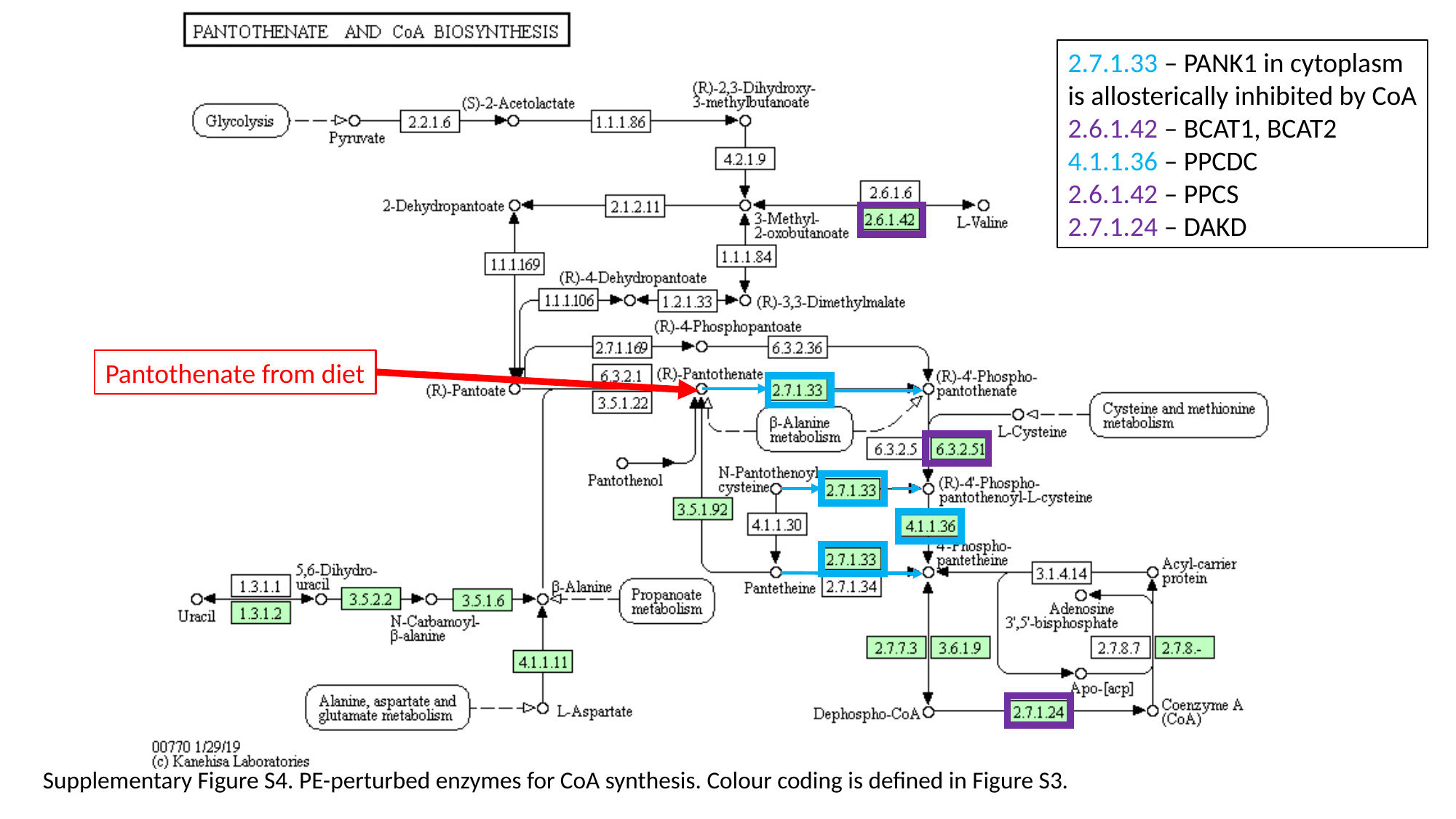

2.7.1.33 – PANK1 in cytoplasm
is allosterically inhibited by CoA
2.6.1.42 – BCAT1, BCAT2
4.1.1.36 – PPCDC
2.6.1.42 – PPCS
2.7.1.24 – DAKD
Pantothenate from diet
Supplementary Figure S4. PE-perturbed enzymes for CoA synthesis. Colour coding is defined in Figure S3.

## Slide 9
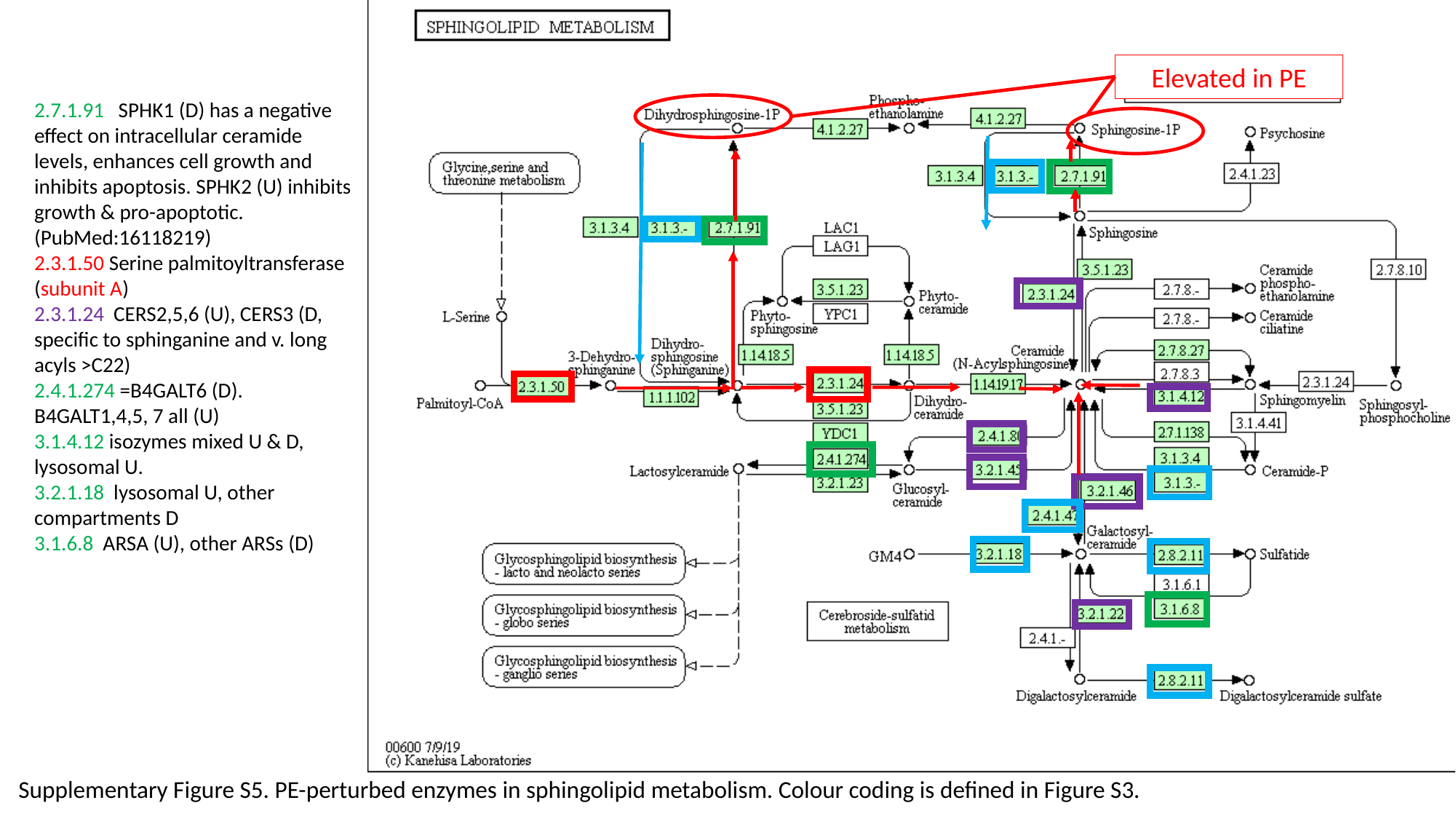

Elevated in PE
2.7.1.91 SPHK1 (D) has a negative effect on intracellular ceramide levels, enhances cell growth and inhibits apoptosis. SPHK2 (U) inhibits growth & pro-apoptotic. (PubMed:16118219)
2.3.1.50 Serine palmitoyltransferase (subunit A)
2.3.1.24 CERS2,5,6 (U), CERS3 (D, specific to sphinganine and v. long acyls >C22)
2.4.1.274 =B4GALT6 (D). B4GALT1,4,5, 7 all (U)
3.1.4.12 isozymes mixed U & D, lysosomal U.
3.2.1.18 lysosomal U, other compartments D
3.1.6.8 ARSA (U), other ARSs (D)
Supplementary Figure S5. PE-perturbed enzymes in sphingolipid metabolism. Colour coding is defined in Figure S3.

## Slide 10
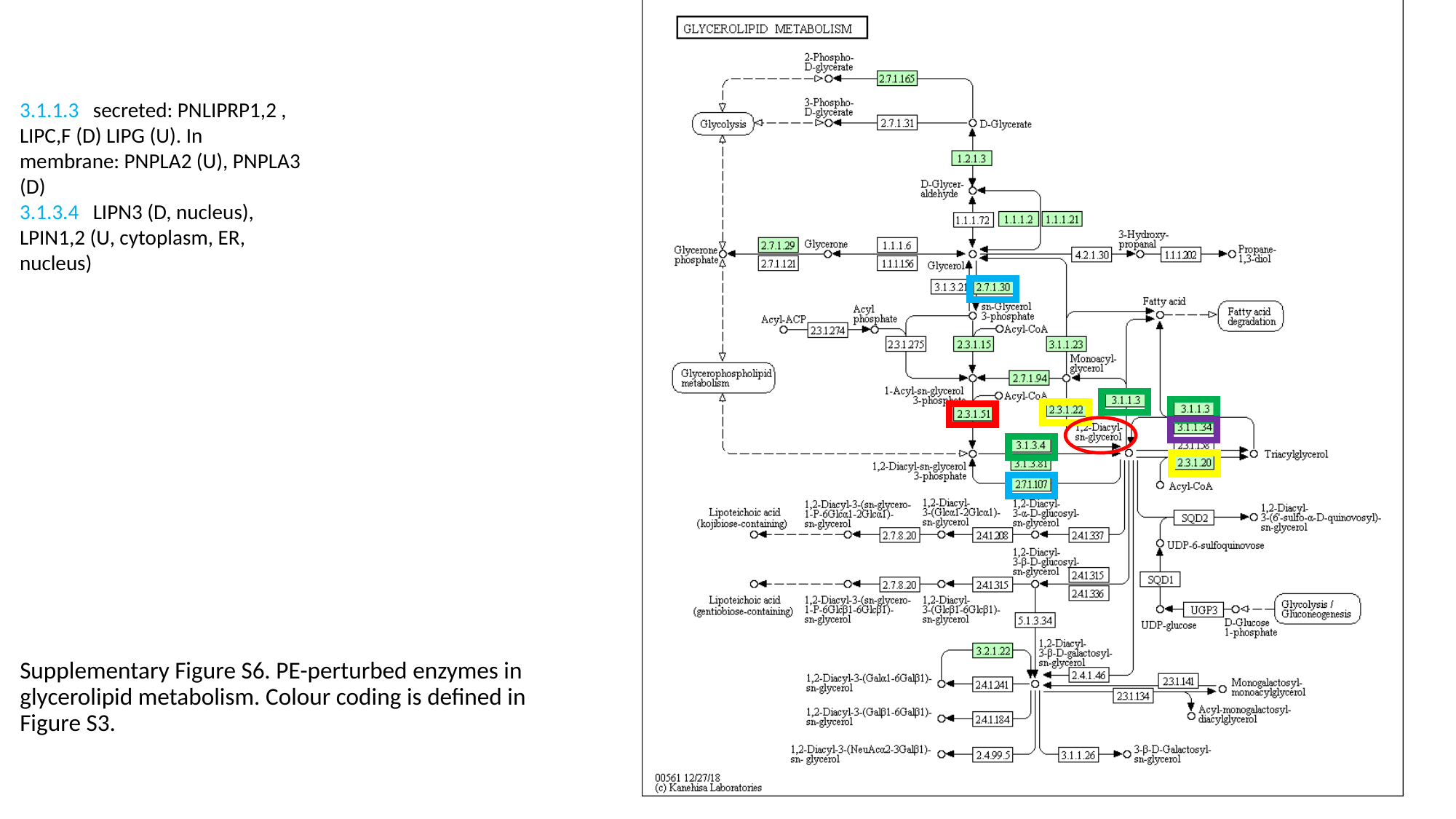

3.1.1.3 secreted: PNLIPRP1,2 , LIPC,F (D) LIPG (U). In membrane: PNPLA2 (U), PNPLA3 (D)
3.1.3.4 LIPN3 (D, nucleus), LPIN1,2 (U, cytoplasm, ER, nucleus)
Supplementary Figure S6. PE-perturbed enzymes in glycerolipid metabolism. Colour coding is defined in Figure S3.

## Slide 11
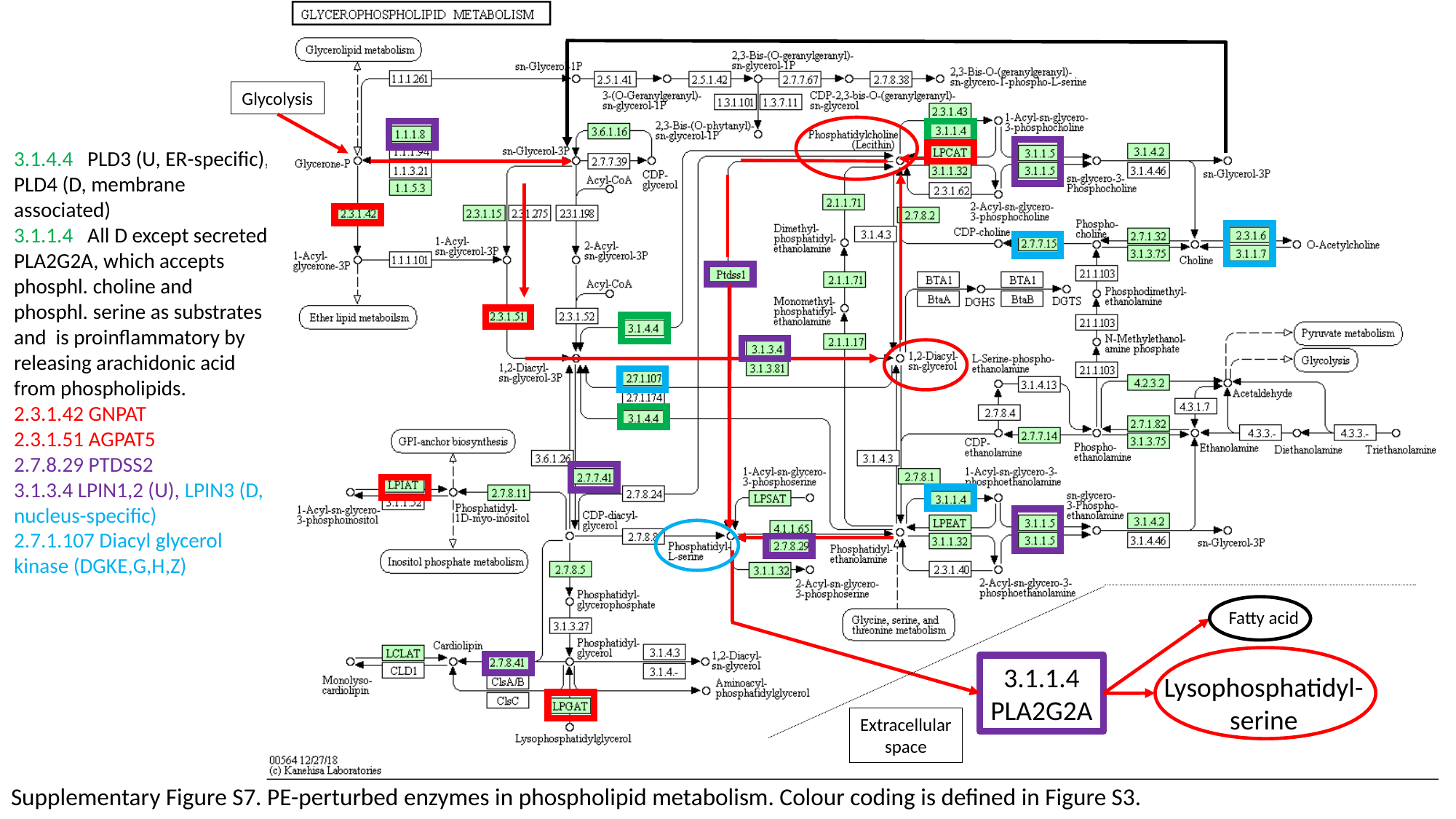

Glycolysis
Fatty acid
3.1.1.4
PLA2G2A
Lysophosphatidyl-
serine
Extracellular
space
3.1.4.4 PLD3 (U, ER-specific), PLD4 (D, membrane associated)
3.1.1.4 All D except secreted PLA2G2A, which accepts phosphl. choline and phosphl. serine as substrates and is proinflammatory by releasing arachidonic acid from phospholipids.
2.3.1.42 GNPAT
2.3.1.51 AGPAT5
2.7.8.29 PTDSS2
3.1.3.4 LPIN1,2 (U), LPIN3 (D, nucleus-specific)
2.7.1.107 Diacyl glycerol kinase (DGKE,G,H,Z)
Supplementary Figure S7. PE-perturbed enzymes in phospholipid metabolism. Colour coding is defined in Figure S3.

## Slide 12
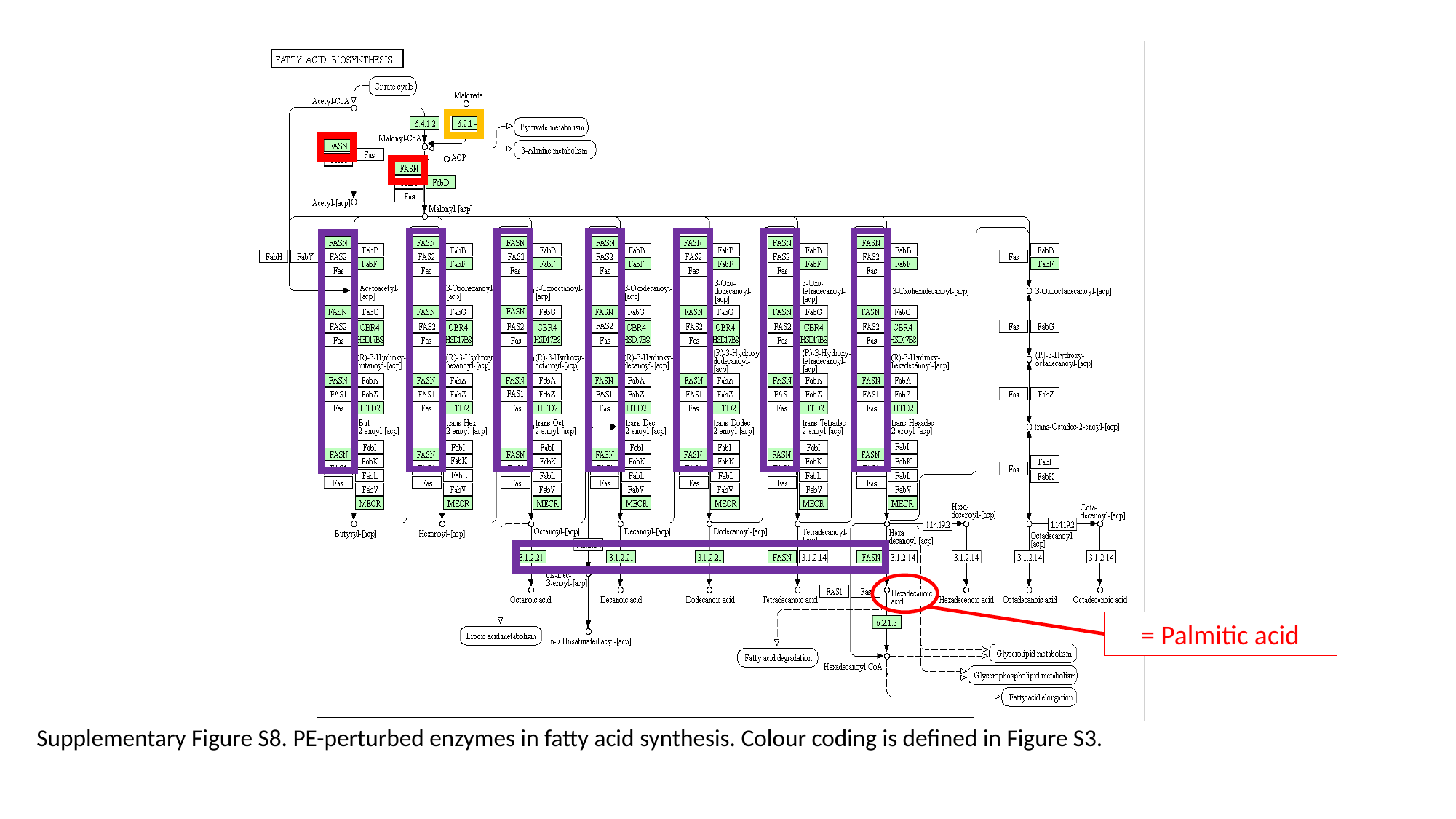

= Palmitic acid
Supplementary Figure S8. PE-perturbed enzymes in fatty acid synthesis. Colour coding is defined in Figure S3.

## Slide 13
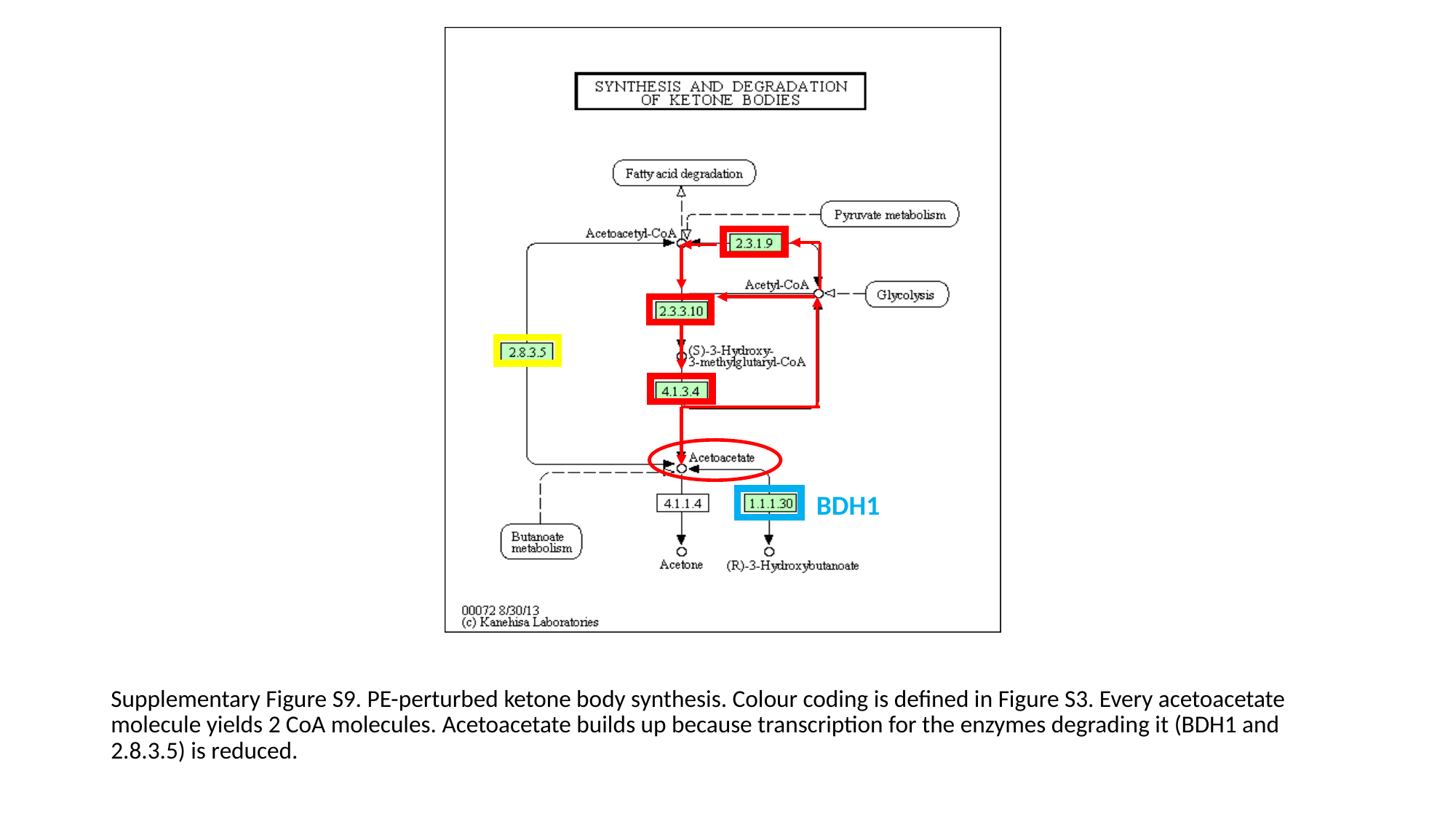

BDH1
Supplementary Figure S9. PE-perturbed ketone body synthesis. Colour coding is defined in Figure S3. Every acetoacetate molecule yields 2 CoA molecules. Acetoacetate builds up because transcription for the enzymes degrading it (BDH1 and 2.8.3.5) is reduced.

## Slide 14
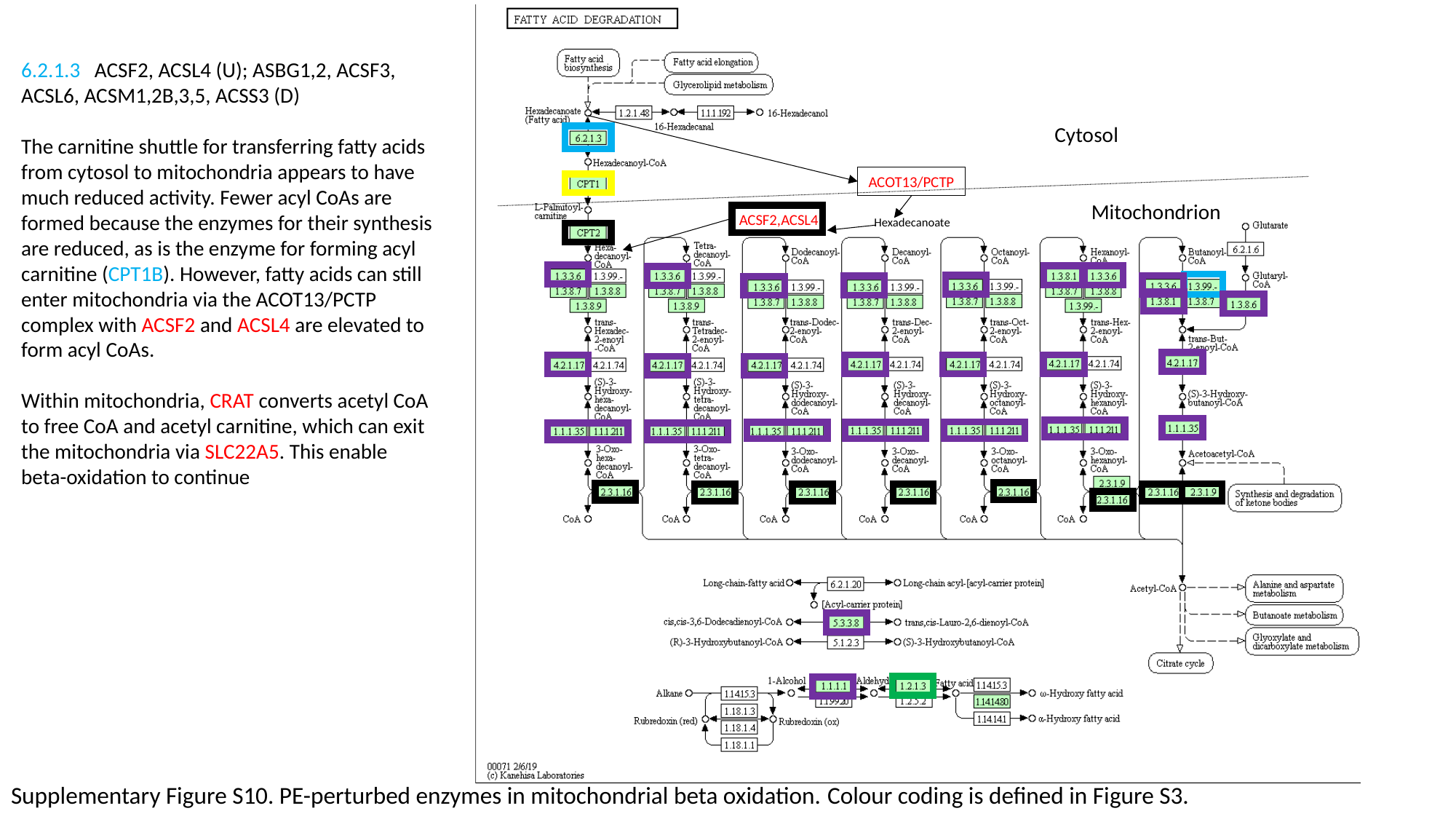

Cytosol
ACOT13/PCTP
Mitochondrion
ACSF2,ACSL4
Hexadecanoate
6.2.1.3 ACSF2, ACSL4 (U); ASBG1,2, ACSF3, ACSL6, ACSM1,2B,3,5, ACSS3 (D)
The carnitine shuttle for transferring fatty acids from cytosol to mitochondria appears to have much reduced activity. Fewer acyl CoAs are formed because the enzymes for their synthesis are reduced, as is the enzyme for forming acyl carnitine (CPT1B). However, fatty acids can still enter mitochondria via the ACOT13/PCTP complex with ACSF2 and ACSL4 are elevated to form acyl CoAs.
Within mitochondria, CRAT converts acetyl CoA to free CoA and acetyl carnitine, which can exit the mitochondria via SLC22A5. This enable beta-oxidation to continue
Supplementary Figure S10. PE-perturbed enzymes in mitochondrial beta oxidation. Colour coding is defined in Figure S3.

## Slide 15
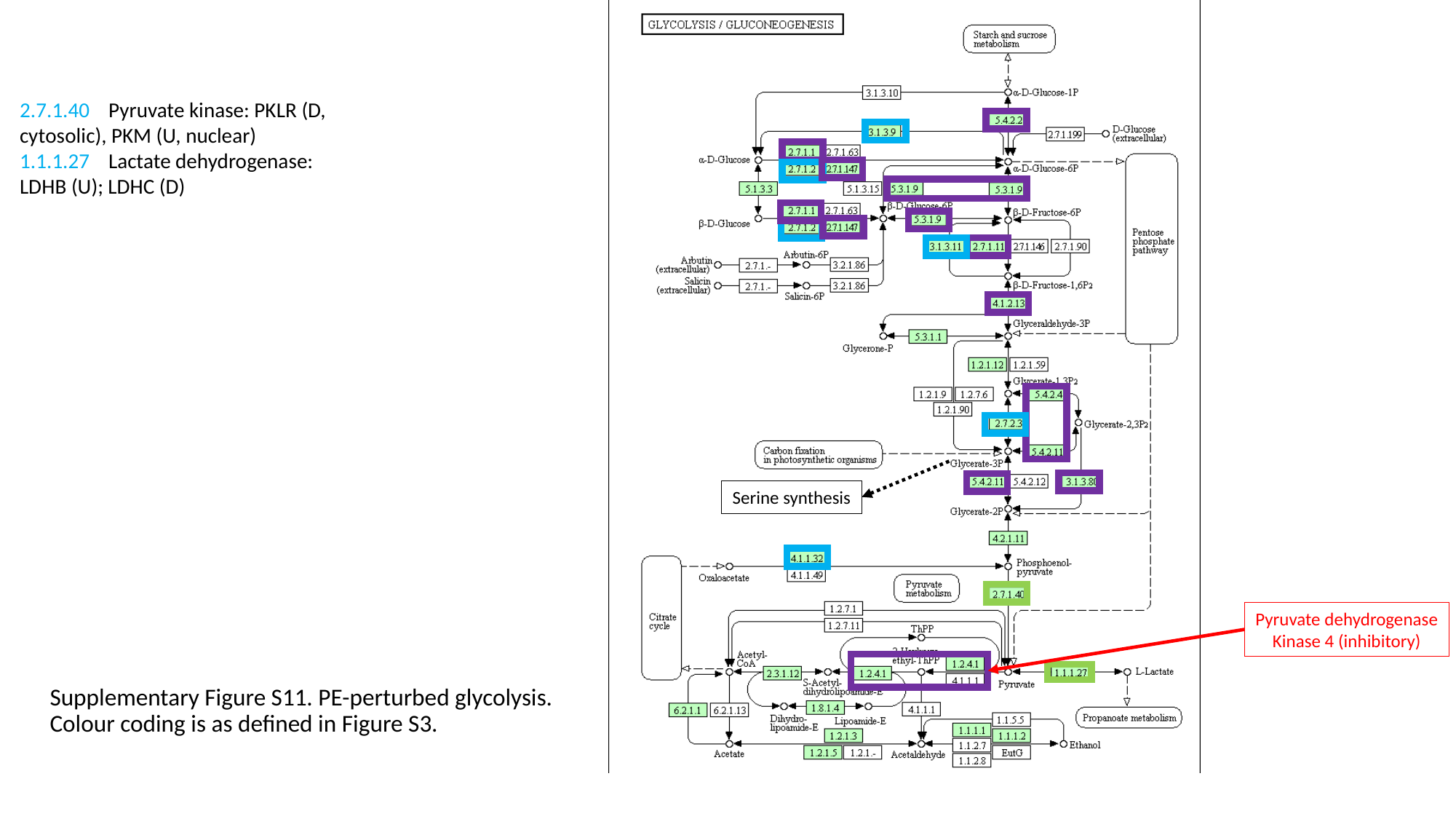

Serine synthesis
Pyruvate dehydrogenase
Kinase 4 (inhibitory)
2.7.1.40 Pyruvate kinase: PKLR (D, cytosolic), PKM (U, nuclear)
1.1.1.27 Lactate dehydrogenase: LDHB (U); LDHC (D)
Supplementary Figure S11. PE-perturbed glycolysis. Colour coding is as defined in Figure S3.

## Slide 16
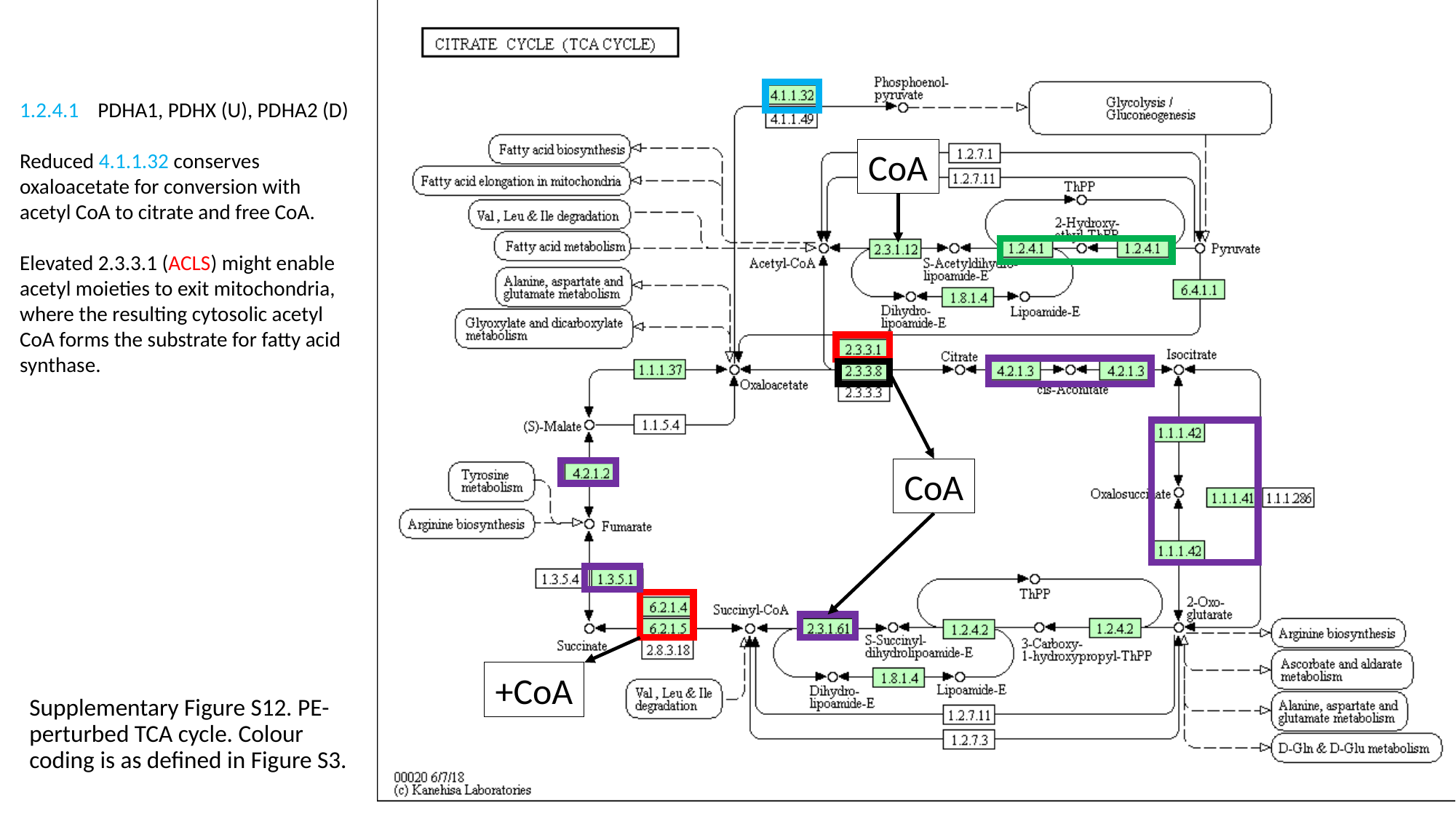

CoA
CoA
+CoA
1.2.4.1 PDHA1, PDHX (U), PDHA2 (D)
Reduced 4.1.1.32 conserves oxaloacetate for conversion with acetyl CoA to citrate and free CoA.
Elevated 2.3.3.1 (ACLS) might enable acetyl moieties to exit mitochondria, where the resulting cytosolic acetyl CoA forms the substrate for fatty acid synthase.
Supplementary Figure S12. PE-perturbed TCA cycle. Colour coding is as defined in Figure S3.

## Slide 17
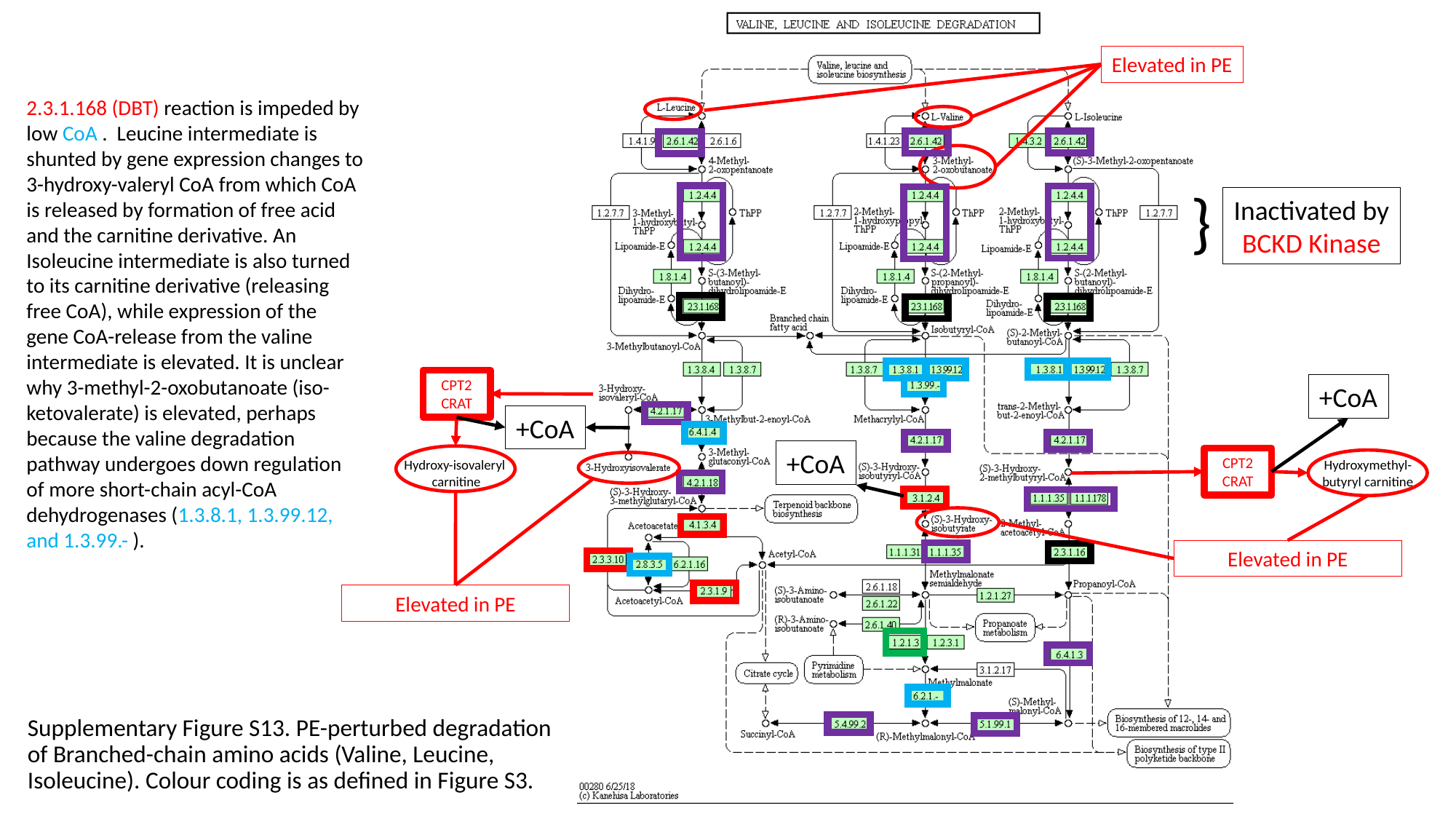

Elevated in PE
}
Inactivated by
BCKD Kinase
CPT2
CRAT
+CoA
+CoA
+CoA
CPT2
CRAT
Hydroxy-isovaleryl
carnitine
Hydroxymethyl-butyryl carnitine
Elevated in PE
Elevated in PE
2.3.1.168 (DBT) reaction is impeded by low CoA . Leucine intermediate is shunted by gene expression changes to 3-hydroxy-valeryl CoA from which CoA is released by formation of free acid and the carnitine derivative. An Isoleucine intermediate is also turned to its carnitine derivative (releasing free CoA), while expression of the gene CoA-release from the valine intermediate is elevated. It is unclear why 3-methyl-2-oxobutanoate (iso-ketovalerate) is elevated, perhaps because the valine degradation pathway undergoes down regulation of more short-chain acyl-CoA dehydrogenases (1.3.8.1, 1.3.99.12, and 1.3.99.- ).
Supplementary Figure S13. PE-perturbed degradation of Branched-chain amino acids (Valine, Leucine, Isoleucine). Colour coding is as defined in Figure S3.

## Slide 18
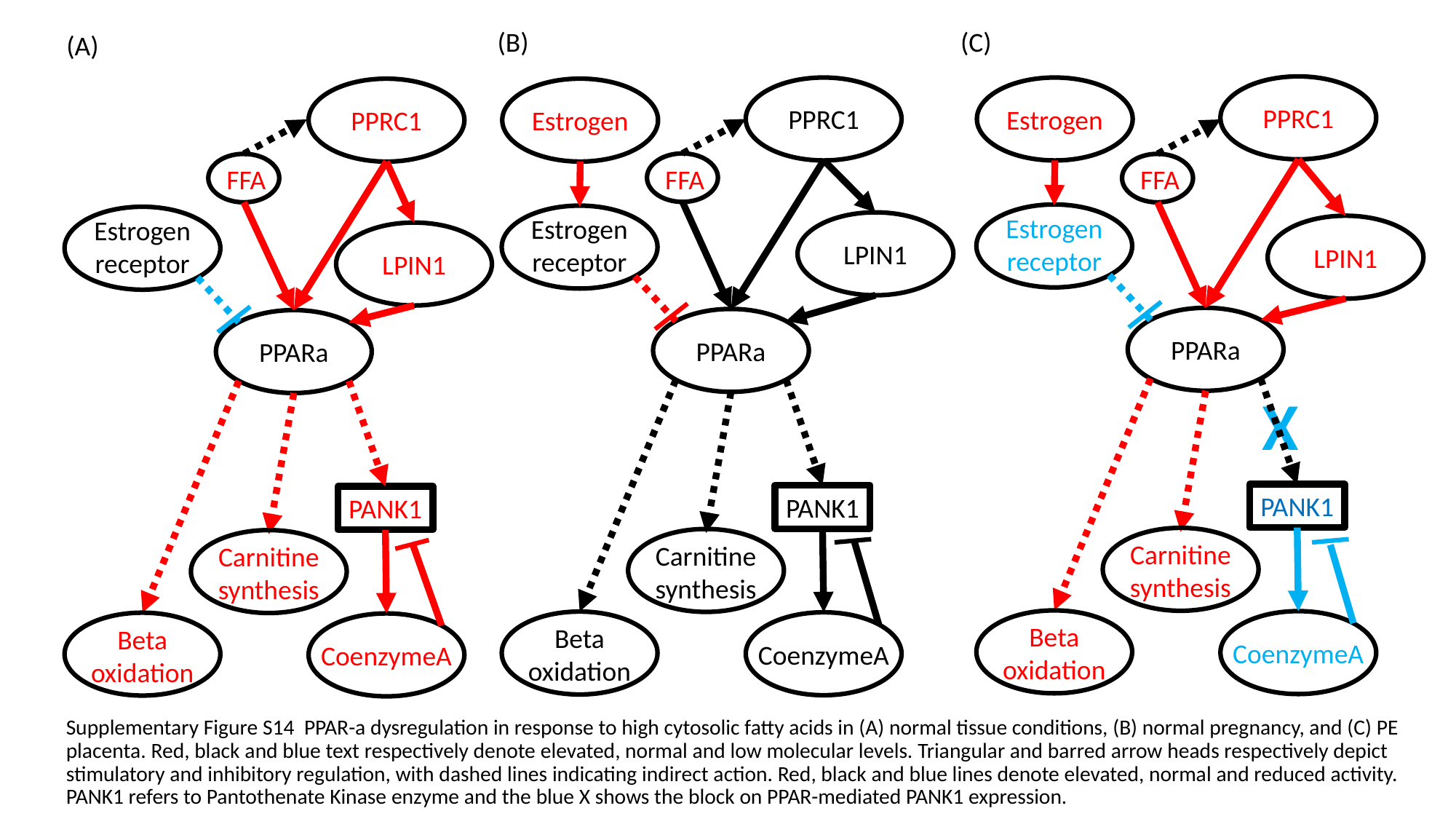

(B)
(C)
(A)
PPRC1
PPRC1
Estrogen
PPRC1
Estrogen
FFA
FFA
FFA
Estrogen
receptor
Estrogen
receptor
Estrogen
receptor
LPIN1
LPIN1
LPIN1
PPARa
PPARa
PPARa
X
PANK1
PANK1
PANK1
Carnitine
synthesis
Carnitine
synthesis
Carnitine
synthesis
Beta
oxidation
Beta
oxidation
Beta
oxidation
CoenzymeA
CoenzymeA
CoenzymeA
Supplementary Figure S14 PPAR-a dysregulation in response to high cytosolic fatty acids in (A) normal tissue conditions, (B) normal pregnancy, and (C) PE placenta. Red, black and blue text respectively denote elevated, normal and low molecular levels. Triangular and barred arrow heads respectively depict stimulatory and inhibitory regulation, with dashed lines indicating indirect action. Red, black and blue lines denote elevated, normal and reduced activity. PANK1 refers to Pantothenate Kinase enzyme and the blue X shows the block on PPAR-mediated PANK1 expression.

## Slide 19
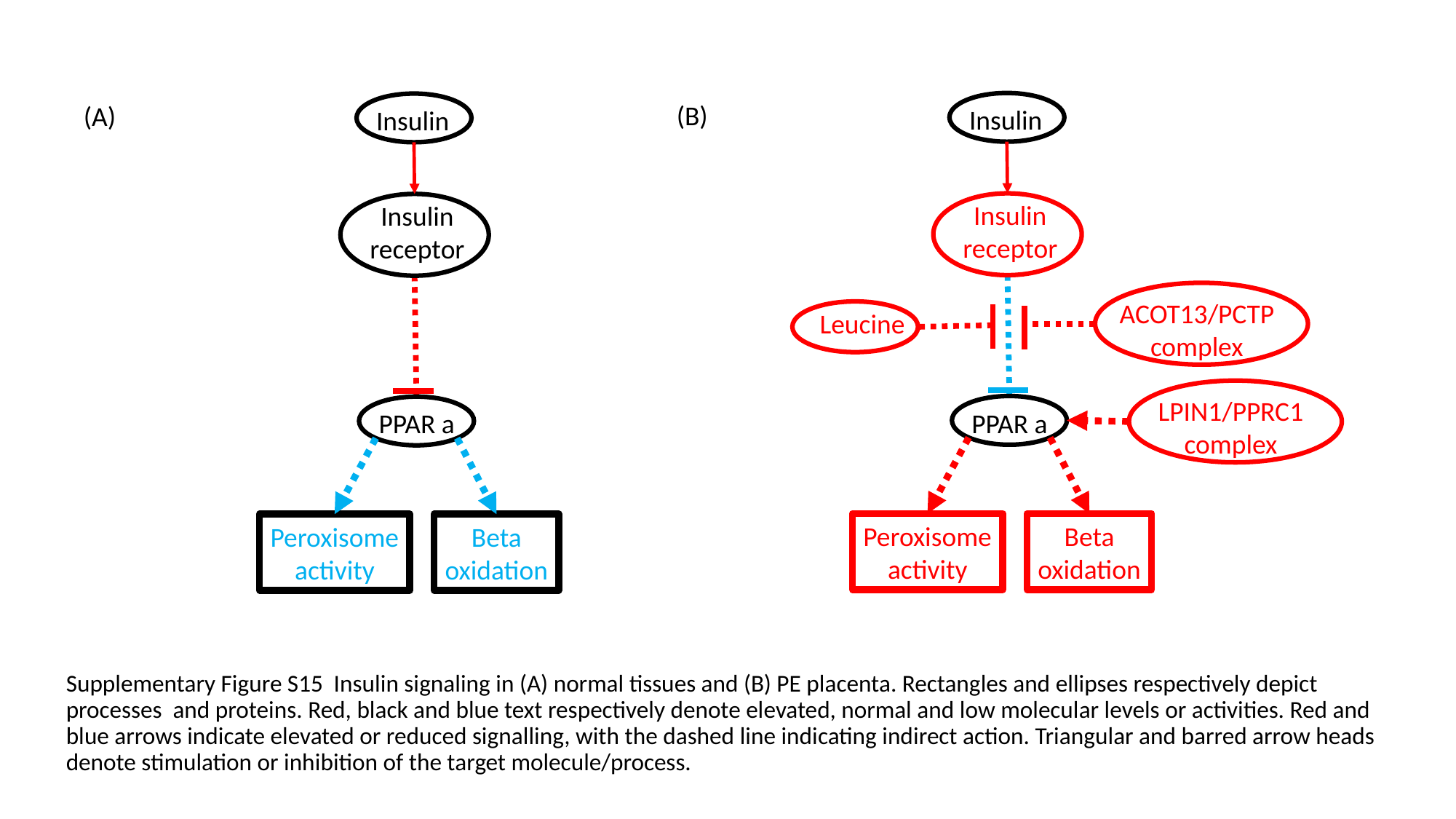

(B)
(A)
Insulin
Insulin
Insulin
receptor
Insulin
receptor
ACOT13/PCTP
complex
Leucine
LPIN1/PPRC1
complex
PPAR a
PPAR a
Peroxisome
activity
Beta
oxidation
Peroxisome
activity
Beta
oxidation
Supplementary Figure S15 Insulin signaling in (A) normal tissues and (B) PE placenta. Rectangles and ellipses respectively depict processes and proteins. Red, black and blue text respectively denote elevated, normal and low molecular levels or activities. Red and blue arrows indicate elevated or reduced signalling, with the dashed line indicating indirect action. Triangular and barred arrow heads denote stimulation or inhibition of the target molecule/process.

## Slide 20
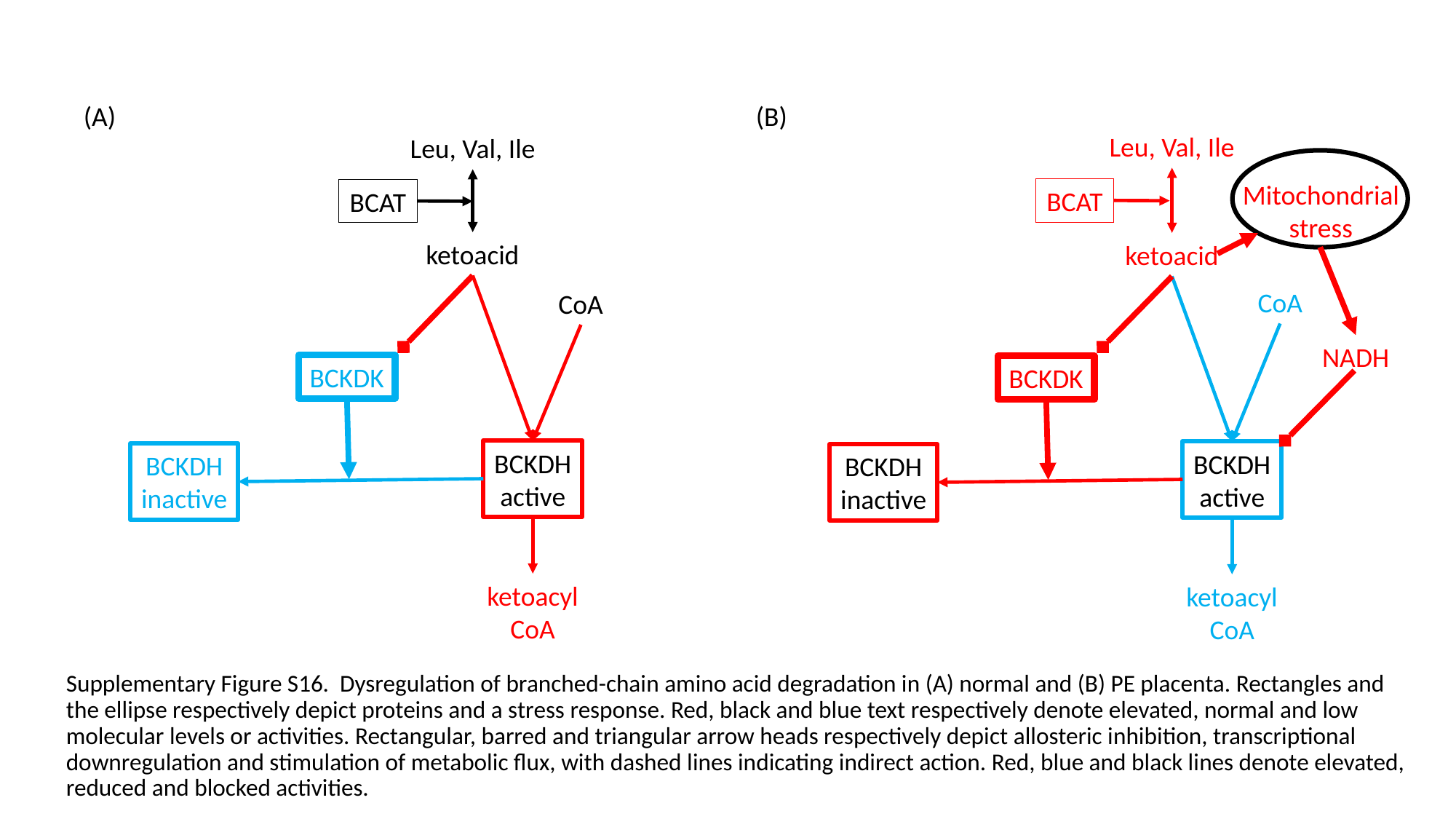

(A)
(B)
Leu, Val, Ile
Leu, Val, Ile
Mitochondrial
stress
BCAT
BCAT
ketoacid
ketoacid
CoA
CoA
NADH
BCKDK
BCKDK
BCKDH
active
BCKDH
active
BCKDH
inactive
BCKDH
inactive
ketoacyl
CoA
ketoacyl
CoA
Supplementary Figure S16. Dysregulation of branched-chain amino acid degradation in (A) normal and (B) PE placenta. Rectangles and the ellipse respectively depict proteins and a stress response. Red, black and blue text respectively denote elevated, normal and low molecular levels or activities. Rectangular, barred and triangular arrow heads respectively depict allosteric inhibition, transcriptional downregulation and stimulation of metabolic flux, with dashed lines indicating indirect action. Red, blue and black lines denote elevated, reduced and blocked activities.

## Slide 21
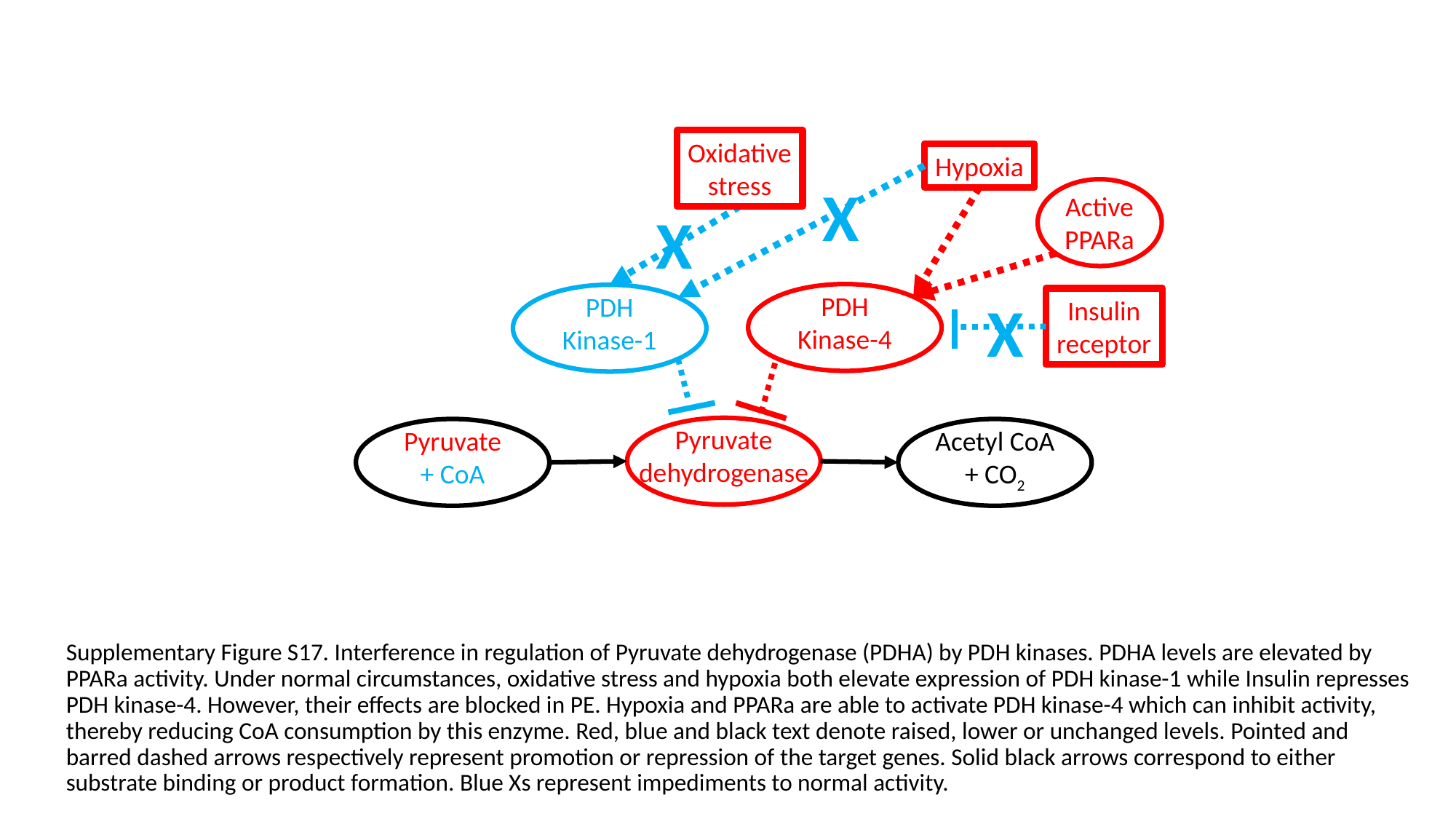

Oxidative
stress
Hypoxia
X
Active
PPARa
X
PDH
Kinase-4
PDH
Kinase-1
X
Insulin
receptor
Pyruvate
dehydrogenase
Pyruvate
+ CoA
Acetyl CoA
+ CO2
Supplementary Figure S17. Interference in regulation of Pyruvate dehydrogenase (PDHA) by PDH kinases. PDHA levels are elevated by PPARa activity. Under normal circumstances, oxidative stress and hypoxia both elevate expression of PDH kinase-1 while Insulin represses PDH kinase-4. However, their effects are blocked in PE. Hypoxia and PPARa are able to activate PDH kinase-4 which can inhibit activity, thereby reducing CoA consumption by this enzyme. Red, blue and black text denote raised, lower or unchanged levels. Pointed and barred dashed arrows respectively represent promotion or repression of the target genes. Solid black arrows correspond to either substrate binding or product formation. Blue Xs represent impediments to normal activity.

## Slide 22
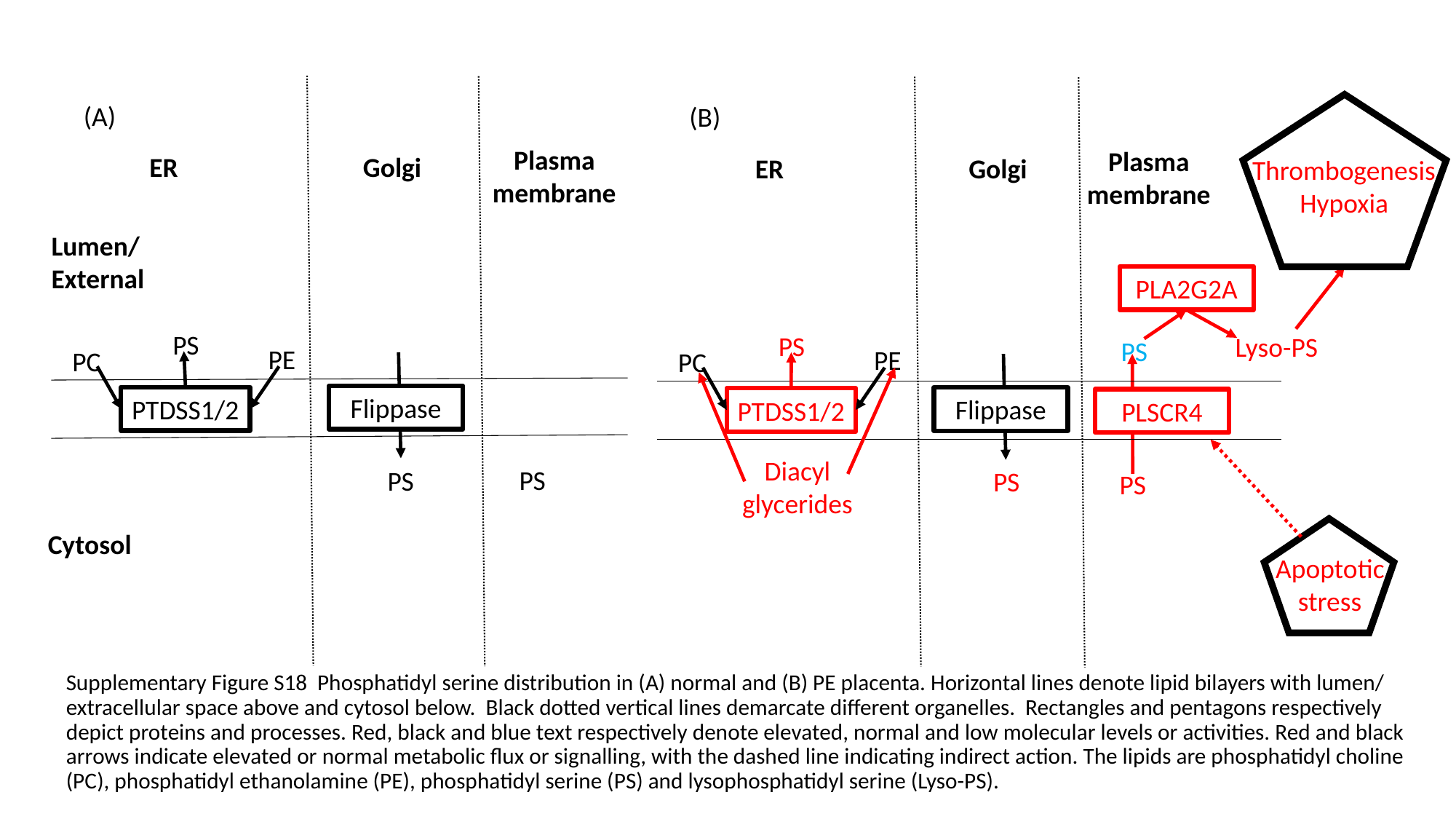

(A)
(B)
Plasma
membrane
Plasma
membrane
ER Golgi
ER Golgi
Thrombogenesis
Hypoxia
Lumen/
External
PLA2G2A
PS
PS
Lyso-PS
PS
PE
PE
PC
PC
Flippase
PTDSS1/2
Flippase
PTDSS1/2
PLSCR4
Diacyl
glycerides
PS
PS
PS
PS
Cytosol
Apoptotic
stress
Supplementary Figure S18 Phosphatidyl serine distribution in (A) normal and (B) PE placenta. Horizontal lines denote lipid bilayers with lumen/ extracellular space above and cytosol below. Black dotted vertical lines demarcate different organelles. Rectangles and pentagons respectively depict proteins and processes. Red, black and blue text respectively denote elevated, normal and low molecular levels or activities. Red and black arrows indicate elevated or normal metabolic flux or signalling, with the dashed line indicating indirect action. The lipids are phosphatidyl choline (PC), phosphatidyl ethanolamine (PE), phosphatidyl serine (PS) and lysophosphatidyl serine (Lyso-PS).

## Slide 23
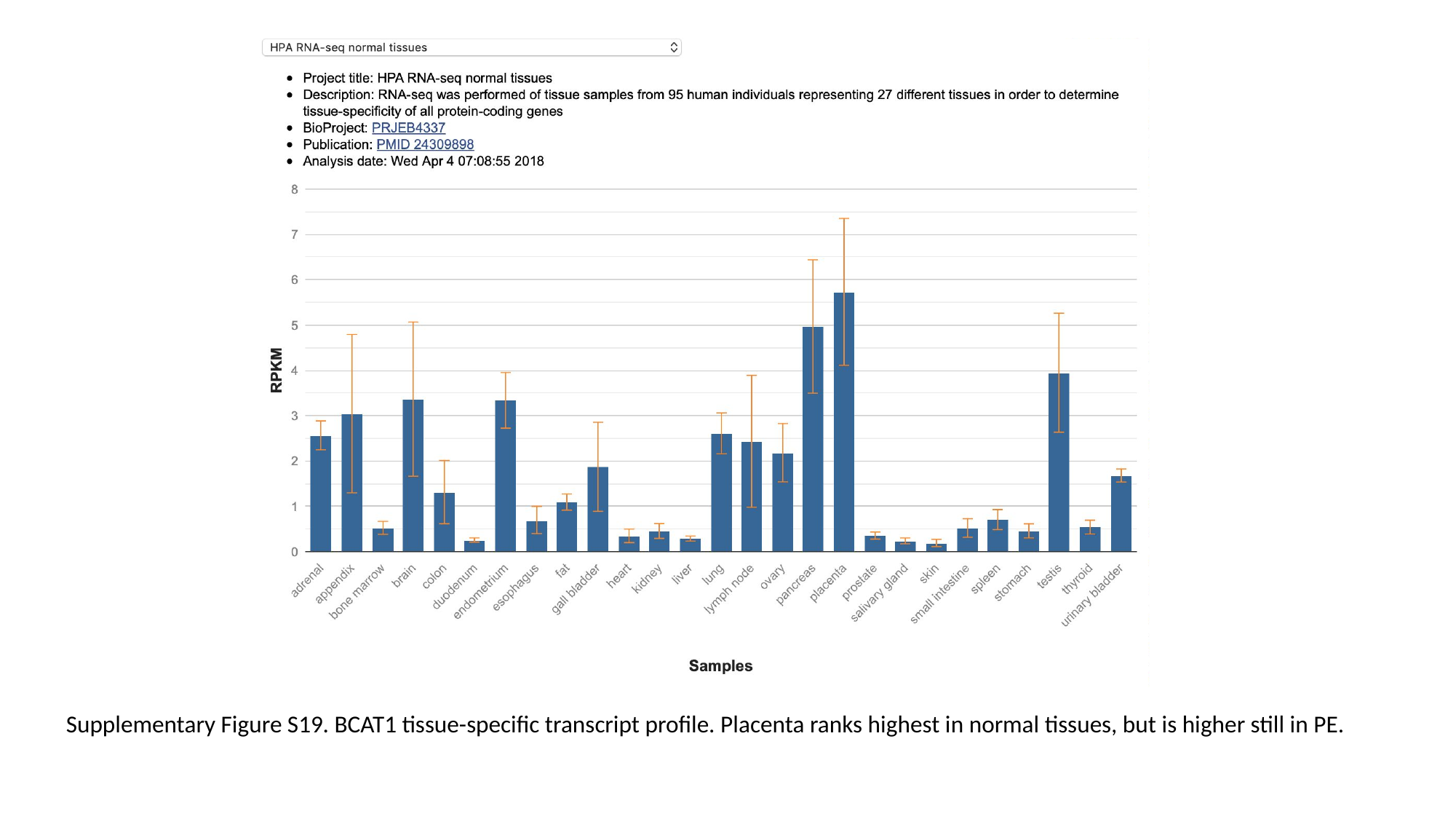

Supplementary Figure S19. BCAT1 tissue-specific transcript profile. Placenta ranks highest in normal tissues, but is higher still in PE.
